# Laser-Engineered Black TiO_2_/UHMWPE Nanocomposites: Tribomechanical Response and Potential for Biomedical Bearing Applications

**DOI:** 10.3390/ma19204384

**Published:** 2026-10-19

**Authors:** Jeidy Negrin-González, Ernesto Lorenzo-Bonet, Osmel Pérez-Acosta, Melvyn Álvarez-Vera, Leonel Díaz-Tato, Sadasivan Shaji, Edgar Omar García-Sánchez, Edén Amaral Rodríguez-Castellanos, Marco Antonio Loudovic Hernández-Rodríguez

**Affiliations:** 1 Universidad Autónoma de Nuevo León (UANL), Facultad de Ingeniería Mecánica y Eléctrica (FIME), San Nicolás de los Garza 66450, Nuevo León, Mexico; jeidy.negringn@uanl.edu.mx (J.N.-G.); ernesto.lorenzobnt@uanl.edu.mx (E.L.-B.); osmel.perezast@uanl.edu.mx (O.P.-A.); leonel.diazat@uanl.edu.mx (L.D.-T.); sadasivan.shaji@uanl.edu.mx (S.S.); edgar.garciasc@uanl.edu.mx (E.O.G.-S.); 2 Departamento de Ingeniería Industrial y Mecánica, Universidad de las Américas Puebla (UDLAP), Ex Hacienda Santa Catarina Mártir, Cholula 72810, Puebla, Mexico; melvyn.alvarez@udlap.mx; 3 Universidad Autónoma de Nuevo León (UANL), Centro de Innovación, Investigación y Desarrollo en Ingeniería y Tecnología (CIIDIT), Parque de Investigación e Innovación Tecnológica (PIIT), Apodaca 66600, Nuevo León, Mexico

**Keywords:** UHMWPE, black TiO_2_, pulsed laser irradiation, nanocomposites, tribology, wear resistance, biomedical bearings, acetabular components

## Abstract

**Highlights:**

- PLIL transformed TiO_2_ toward a rounded morphology and a rutile-dominant phase composition. The term “black TiO_2_” is an operational description for PLIL-irradiated powder; defect-related chemical characterizations were not performed in this work.
- At 0.5 wt%, black TiO_2_ exhibited the lowest mean wear volume, although the reduction versus neat UHMWPE was not statistically significant.
- UHMWPE wear resistance depended on TiO_2_ state and loading rather than maximum hardness or minimum friction.

**Abstract:**

Wear of ultra-high-molecular-weight polyethylene (UHMWPE) remains a limitation for long-term orthopedic bearing performance. This study investigated how pulsed laser irradiation in liquid (PLIL)-induced modification of TiO_2_ affects the mechanical and tribological response of UHMWPE nanocomposites containing 0.1–1.0 wt% TiO_2_. PLIL changed TiO_2_ from predominantly irregular/angular particles with an anatase-dominant composition (~56% anatase/44% rutile) to a greater prevalence of rounded particles with a rutile-dominant balance (~41% anatase/59% rutile). TiO_2_ incorporation produced a statistically significant but moderate microhardness increase, reaching 4.19 ± 0.12 HV0.05 at 1.0 wt% untreated TiO_2_, approximately 25% above neat UHMWPE. Tribological behavior was formulation dependent: 0.5 wt% PLIL-modified black TiO_2_ exhibited the lowest mean wear volume (2.06 ± 0.02 mm^3^), 13.1% below neat UHMWPE (2.37 ± 0.23 mm^3^), although the difference was not statistically significant (*p* = 0.739). Maximum microhardness, minimum terminal friction, and minimum mean wear occurred in different formulations, demonstrating that these responses are partially decoupled. Nanoparticle state and concentration jointly governed the tribomechanical response within the investigated low-filler regime.

## 1. Introduction

Ultra-high-molecular-weight polyethylene (UHMWPE) is one of the most widely used polymeric bearing materials in total joint arthroplasty because of its favorable combination of toughness, low friction, wear resistance, chemical stability, and biocompatibility [1,2,3,4,5,6]. Its exceptionally high molecular weight and highly entangled semicrystalline structure provide substantial resistance to impact, plastic deformation, and surface damage, making UHMWPE particularly suitable for articulating bearing components [1,3]. Nevertheless, despite its long-standing clinical success, wear remains an important materials-related limitation affecting the long-term performance of UHMWPE. Repeated sliding and cyclic contact can generate polymeric wear particles capable of triggering inflammatory responses in periprosthetic tissues and promoting osteoclast-mediated bone resorption, thereby contributing to osteolysis, aseptic loosening, and eventual loss of implant stability [7,8,9]. Consequently, reducing wear while preserving the mechanical integrity of UHMWPE remains a central challenge in the development of more durable polymeric bearing materials.

Different strategies have been investigated to enhance the mechanical and tribological response of UHMWPE, including modifications of the polymer structure, surface engineering, and reinforcement with micro- and nanoscale fillers [4,7,10,11,12,13,14,15,16]. Among these approaches, nanocomposite reinforcement is particularly attractive because relatively small filler fractions can modify resistance to local deformation, surface hardness, load transfer, and the evolution of the sliding interface without requiring major changes in the bulk polymer composition. However, nanoparticle incorporation does not inherently guarantee improved tribological performance. Its effectiveness depends strongly on filler concentration, particle size and morphology, dispersion, matrix–particle adhesion, and the ability of the reinforcement to remain embedded within the polymer during sliding [10,11,12,13,14,16]. Well-dispersed rigid nanoparticles may restrict local deformation and contribute to load support, whereas agglomeration, weak interfacial bonding, or particle pull-out may generate additional debris and promote abrasive or three-body wear. In addition, the formation and stability of transfer films on the counterface can substantially influence the friction and wear response of polymer composites, with nanoparticles potentially contributing either to protective transfer layers or to abrasive interactions depending on the tribological conditions [17,18]. Accordingly, the tribological response of particle-reinforced UHMWPE may exhibit a non-monotonic dependence on filler content, making optimization of both nanoparticle physicochemical state and concentration essential for rational nanocomposite design.

Titanium dioxide (TiO_2_) represents an attractive ceramic nanofiller for polymer-based biomedical materials because of its chemical stability, mechanical robustness, corrosion resistance, and extensive investigation in biologically relevant systems [19,20]. TiO_2_ has also received considerable attention for photocatalytic and antimicrobial applications, although these functionalities depend strongly on crystalline phase, particle size, surface chemistry, defect structure, and environmental conditions [19,20]. In the present context, however, the principal interest of TiO_2_ lies in its ability to modify the mechanical response of UHMWPE and the interactions developing at the sliding interface. Recent work has further demonstrated the biomedical potential of TiO_2_-containing UHMWPE-based composites, with Salama et al. reporting that HAp/TiO_2_ reinforcement can influence wear, fatigue, and antibacterial performance [16]. More broadly, tribological studies of TiO_2_-reinforced polymer composites have shown that the effect of TiO_2_ is strongly dependent on the tribological system, including its ability to participate in the formation of nanoparticle-enriched transfer films at the sliding interface [17]. Previous investigations involving titanium-containing particles, inorganic fillers, and carbonaceous nanomaterials have demonstrated that reinforcement can modify hardness, friction, and wear behavior in UHMWPE, although the magnitude and even the direction of these effects are strongly dependent on filler characteristics, dispersion, interfacial adhesion, and concentration [4,10,11,12,13,14,21,22].

The concentration of the nanofiller is particularly important because the balance between effective reinforcement and particle-induced defects may occur over a relatively narrow loading interval. Previous UHMWPE nanocomposite studies have demonstrated measurable changes in mechanical and tribological properties at relatively low nanoparticle concentrations [14,16,21]. Recent TiO_2_-containing UHMWPE studies further indicate that the tribomechanical response may be non-monotonic with filler content, with intermediate TiO_2_ concentrations potentially providing more favorable performance than higher loadings where particle agglomeration can become increasingly important [16]. This consideration is particularly relevant for nanoscale TiO_2_ because its high specific surface area generates a substantial polymer–particle interfacial area even at relatively low mass fractions. Consequently, increasing the nominal nanoparticle content does not necessarily produce a proportional increase in effective reinforcement. As the particle population increases, particle–particle interactions and agglomeration may become increasingly important, potentially generating particle-rich heterogeneities and weakly bonded regions and reducing the fraction of effectively dispersed reinforcement available for interfacial load transfer. Under sliding contact, insufficiently retained particles or agglomerates may additionally become detached from the polymer matrix and participate in third-body abrasive interactions, thereby potentially offsetting the mechanical benefits provided by the ceramic reinforcement. Thus, the relationship between TiO_2_ content and wear resistance should not be expected to follow a simple monotonic “higher filler–greater reinforcement” behavior.

On this basis, the present investigation deliberately focuses on the 0.1–1.0 wt% TiO_2_ interval as a low-filler experimental regime rather than attempting to determine the maximum TiO_2_ content that can be incorporated into UHMWPE. The upper boundary of 1.0 wt% was selected to investigate concentration-dependent reinforcement within this low-loading regime while limiting the increasing probability of dispersion- and interface-related effects associated with a progressively larger nanoparticle population. The selected concentrations of 0.1, 0.3, 0.5, 0.8, and 1.0 wt% provide sufficient experimental resolution to determine whether an intermediate reinforcement level produces a more favorable balance among mechanical strengthening, friction, and wear resistance than either very low or comparatively higher nanoparticle contents [23]. Accordingly, 1.0 wt% should be interpreted as the upper boundary of the deliberately selected low-filler regime investigated in this study, rather than as a proposed global optimum or maximum useful TiO_2_ concentration. TiO_2_ contents above 1.0 wt% remain outside the experimental scope of the present investigation and warrant systematic evaluation in future studies.

Beyond filler concentration, deliberately tailoring the physicochemical state of TiO_2_ before its incorporation into UHMWPE offers an additional strategy for controlling nanocomposite performance. Conventional white TiO_2_ generally contains anatase and/or rutile crystalline phases, whereas black TiO_2_ is a broad term encompassing dark-colored TiO_2_ materials whose physicochemical characteristics depend strongly on the synthesis route [19,20,24,25,26]. Black TiO_2_ should therefore not be regarded as a distinct crystallographic phase or merely as an optical modification of conventional TiO_2_. Depending on the processing method, its dark appearance and altered properties have been associated with oxygen deficiency, Ti^3+^ states, surface disorder, modified electronic states, changes in crystallinity and phase composition, or combinations of these features [24,25,26]. Importantly, the presence and relative contribution of these characteristics must be established experimentally for each specific material system and should not be inferred solely from the dark appearance of the material. In the present study, the term “PLIL-modified black TiO_2_” is therefore used operationally to describe the visibly darkened TiO_2_ powder obtained after pulsed laser irradiation in liquid. This terminology does not imply experimentally verified Ti^3+^ species, oxygen vacancies, surface disorder, or a specific electronic defect structure. The experimentally demonstrated PLIL-induced modifications considered in the present work are restricted primarily to changes in nanoparticle morphology and anatase/rutile crystalline-phase balance.

Several chemical and physical approaches have been investigated to modify TiO_2_, including reduction treatments, hydrothermal and thermal processing, and laser-assisted methods [19,20,27,28,29]. Pulsed laser processing in liquids is particularly attractive because it can reduce the need for chemical reagents while providing control over nanoparticle characteristics through variables such as wavelength, pulse duration, pulse energy, and irradiation time [27,28,29]. When pre-existing nanoparticles dispersed in a liquid are directly exposed to pulsed laser radiation, the process is more specifically described as pulsed laser irradiation in liquid (PLIL). Previous investigations have demonstrated that PLIL can produce pronounced morphological restructuring of TiO_2_ nanoparticles together with modifications in the relative fractions of anatase and rutile [29]. These changes have been associated with rapid laser-energy absorption and localized thermal processes capable of promoting particle restructuring and phase transformation. From a tribological perspective, such laser-induced modifications are potentially important because particle morphology and crystalline state may influence local contact mechanics, stress transfer, particle retention within the polymer matrix, and the development of the sliding interface.

Despite extensive investigations of UHMWPE reinforced with nanofillers [11,12,21,22] and previous demonstrations of black TiO_2_ production by pulsed laser irradiation in liquid [29], the relationship between PLIL-induced modification of TiO_2_ and the resulting tribomechanical response of TiO_2_/UHMWPE nanocomposites remains insufficiently established. More specifically, to the best of our knowledge, a systematic direct comparison between untreated white TiO_2_ and visibly darkened PLIL-modified TiO_2_, operationally referred to here as black TiO_2_, incorporated into the same UHMWPE matrix, across the same low-concentration range and under otherwise identical processing and tribological conditions, has not been reported. This distinction is important because nanoparticles with nominally identical chemical composition and concentration may generate markedly different reinforcement and wear responses when their morphology and crystalline-phase balance are modified. Establishing such a comparison therefore provides an opportunity to evaluate the influence of the experimentally observed PLIL-induced physicochemical modifications of the TiO_2_ reinforcement independently of filler concentration.

Accordingly, the present study systematically compares UHMWPE reinforced with untreated white TiO_2_ and PLIL-modified black TiO_2_ nanoparticles at concentrations of 0.1, 0.3, 0.5, 0.8, and 1.0 wt%. Particle morphology and crystalline structure were characterized to establish the modifications produced by laser irradiation, while the resulting UHMWPE nanocomposites were evaluated through spectroscopic analysis, Vickers microhardness, instrumented-indentation load–displacement analysis, and dry ball-on-disc tribological testing. It was hypothesized that the experimentally observed PLIL-induced changes in TiO_2_ morphology and anatase/rutile phase balance would be associated with measurable differences in the concentration-dependent mechanical and tribological responses of the resulting UHMWPE nanocomposites relative to those containing untreated white TiO_2_. Particular attention was therefore directed toward determining how nanoparticle state and concentration influence the balance among Vickers microhardness, friction, and wear rather than assuming that a single formulation would simultaneously optimize all three responses.

The dry ball-on-disc experiments employed in this study are intended as a controlled comparative materials-screening methodology rather than as a direct simulation of physiological joint operation. More application-specific evaluation of orthopedic bearing materials requires joint-simulator approaches capable of incorporating clinically relevant articulation, loading, multidirectional motion, and lubricating environments [30,31,32]. Accordingly, the present work does not seek to establish in-service performance of an acetabular component, but rather to determine fundamental processing–structure–property relationships among laser modification, TiO_2_ physicochemical state, filler concentration, mechanical response, friction, and wear under reproducible dry-sliding conditions. By isolating these variables, the study provides a materials-science basis for identifying promising TiO_2_/UHMWPE formulations that may subsequently warrant evaluation under more application-specific biotribological conditions.

## 2. Materials and Methods

### 2.1. Raw Materials

Ultra-high-molecular-weight polyethylene (UHMWPE) powder was purchased from Sigma-Aldrich (Sigma-Aldrich, Merck, Darmstadt, Germany; product No. 429015) and used without additives. According to the supplier specifications, the UHMWPE had a molecular weight of 3–6 × 10^6^ g mol^−1^, an average particle size of 140 ± 20 μm, and a density of 0.94 g cm^−3^. Commercial white TiO_2_ nanoparticles (SkySpring Nanomaterials, Inc., Houston, TX, USA; product No. 7918DL; nominal purity: 99.5%) with a nominal D_50_ particle size of 10–30 nm were used as the starting ceramic reinforcement. According to the supplier specifications, the TiO_2_ nanopowder consisted of a mixture of anatase (60–70 wt%) and rutile (30–40 wt%), with a true density of 4.23 g cm^−3^ and a specific surface area of 50–100 m^2^ g^−1^.

Deionized water (CTR Scientific, Monterrey, Mexico) was used as the liquid medium for pulsed laser irradiation. Analytical-grade acetone (CTR Scientific, Monterrey, Mexico) was used for nanoparticle dispersion and subsequent mixing with UHMWPE.

### 2.2. Preparation of PLIL-Modified Black TiO_2_ Nanoparticles

Commercial white TiO_2_ nanoparticles were subjected to pulsed laser irradiation in liquid (PLIL) as described below. A suspension containing 0.15 g of white TiO_2_ nanoparticles in 10 mL of deionized water was initially homogenized by bath ultrasonication (CS-UB32, CScientific, Mexico City, Mexico) for 15 min at 40 kHz. The ultrasonic bath had a maximum adjustable ultrasonic power of 120 W. The homogenized suspension was subsequently transferred to a 20 mL borosilicate-glass vial for laser irradiation.

PLIL treatment was performed using a Q-switched Nd:YAG laser (LQ629, Solar Laser Systems, Minsk, Belarus) operating at its second-harmonic wavelength of 532 nm, with a pulse duration of 10 ns, a repetition rate of 10 Hz, and a pulse energy of 350 mJ. The laser beam was focused using a plano-convex lens with a focal length of 100 mm. The optical configuration and irradiation geometry were maintained constant throughout the experiments, and the beam position relative to the liquid surface was kept constant during irradiation. At the suspension irradiation plane, the laser spot had a measured diameter of 8.3 mm, corresponding to a beam area of 0.54 cm^2^. The nominal laser fluence was calculated from the pulse energy divided by the beam area at the irradiation plane and was approximately 0.65 J cm^−2^ [29].

The TiO_2_ suspension was irradiated for 90 min at 10 Hz, corresponding to approximately 54,000 laser pulses. During irradiation, the suspension was continuously agitated by magnetic stirring at approximately 300 rpm to minimize nanoparticle sedimentation and promote exposure of the suspended particles to the laser beam. The suspension temperature was continuously monitored using a K-type thermocouple connected to a digital temperature logger (HH506RA, OMEGA Engineering, Inc., Norwalk, CT, USA). The thermocouple probe was immersed in the suspension and positioned near the vial wall, outside the direct laser-beam path. The irradiation vial was placed in a temperature-controlled water bath connected to a recirculating chiller (CORIO CD, JULABO GmbH, Seelbach, Germany), which was set to maintain the suspension temperature at 25 ± 2 °C throughout the 90 min irradiation period.

Three PLIL treatments were independently performed under the same nominal irradiation conditions. However, no batch-resolved quantitative FESEM, XRD, Raman, or other physicochemical characterization was performed independently for the three irradiated preparations. Accordingly, these preparations were not used to establish quantitative batch-to-batch reproducibility, and no such reproducibility claim is made in the present study.

After irradiation, each suspension was dried at 60 °C until the aqueous phase was completely removed. The recovered PLIL-treated powder exhibited a visibly dark coloration relative to the untreated white TiO_2_ precursor and is therefore referred to throughout this work as “PLIL-modified black TiO_2_.” This designation is used as an operational descriptor of the treated material and does not imply experimentally established Ti^3+^ content, oxygen-vacancy concentration, surface disorder, or a specific electronic defect structure, because defect-sensitive chemical or optical analyses such as X-ray photoelectron spectroscopy (XPS), electron paramagnetic resonance (EPR), or UV–Vis diffuse-reflectance spectroscopy were not performed in the present study.

For the formulations containing PLIL-modified black TiO_2_, each independently fabricated composite replicate was prepared using TiO_2_ obtained from a different independently irradiated PLIL preparation. Thus, the three composite replicates incorporated independent PLIL-treatment histories as well as independent composite mixing and fabrication steps.

### 2.3. Preparation of TiO_2_/UHMWPE Nanocomposites

Untreated white TiO_2_ and PLIL-modified black TiO_2_ were separately incorporated into UHMWPE at concentrations of 0.1, 0.3, 0.5, 0.8, and 1.0 wt%. As discussed in Section 1, these concentrations were selected to systematically interrogate the low-filler nanocomposite regime and to resolve possible non-monotonic concentration-dependent effects, rather than to establish the maximum TiO_2_ loading achievable in UHMWPE. For the 2.50 g batches used in this study, the nominal UHMWPE/TiO_2_ masses were 2.4975/0.0025, 2.4925/0.0075, 2.4875/0.0125, 2.4800/0.0200, and 2.4750/0.0250 g for the 0.1, 0.3, 0.5, 0.8, and 1.0 wt% formulations, respectively. The same nominal TiO_2_ mass was used for the corresponding untreated white-TiO_2_ and PLIL-modified black-TiO_2_ formulations.

For each formulation, the corresponding amount of untreated white or PLIL-modified black TiO_2_ nanoparticles was dispersed in acetone at a fixed concentration of 1 mg mL^−1^. Accordingly, 2.5, 7.5, 12.5, 20.0, and 25.0 mg of TiO_2_ were dispersed in 2.5, 7.5, 12.5, 20.0, and 25.0 mL of acetone, respectively, for the 0.1, 0.3, 0.5, 0.8, and 1.0 wt% formulations. Each nanoparticle suspension was subjected to bath sonication at 40 kHz for 30 min at room temperature.

The corresponding amount of UHMWPE powder was separately suspended in acetone and subsequently combined with the sonicated TiO_2_ suspension. The resulting TiO_2_/UHMWPE suspension was homogenized by magnetic stirring at 400 rpm for 30 min at room temperature to promote nanoparticle dispersion throughout the polymer suspension. After homogenization, magnetic stirring was continued during solvent evaporation until the acetone was completely removed. The resulting TiO_2_/UHMWPE powder mixture was subsequently dried at 60 °C for 24 h to remove residual solvent and moisture prior to hot pressing.

The dried powder mixtures were consolidated by hot pressing in a cylindrical die with an internal diameter of 30 mm. The powder-filled die was heated from room temperature to 180 °C at a heating rate of 10 °C min^−1^. Once the target temperature was reached, a nominal pressure of 15 MPa was applied and maintained for 15 min at 180 °C. Following the isothermal consolidation stage, the specimens were cooled under pressure to approximately 25 °C, with the applied pressure maintained at 15 MPa throughout the cooling stage. The cooling process required approximately 5 min. Maintaining the pressure during cooling was intended to promote dimensional stability and minimize shrinkage or warpage during solidification. Once approximately 25 °C was reached, the pressure was released and the consolidated specimens were removed from the die.

The resulting disk-shaped specimens had nominal dimensions of 30 mm in diameter and 4 mm in thickness. Neat UHMWPE specimens were fabricated using the same mixing, drying, and hot-pressing conditions as those applied to the TiO_2_-containing composites.

For experimental replication, three independently prepared and fabricated disks (*n* = 3) were produced for each material condition, with each disk originating from an independently prepared TiO_2_/UHMWPE powder mixture. For the formulations containing PLIL-modified black TiO_2_, the relationship between the independently irradiated PLIL preparations and the composite replicates was as specified in Section 2.2.

The experimental design comprised 11 material conditions: neat UHMWPE, five UHMWPE formulations containing untreated white TiO_2_ (0.1, 0.3, 0.5, 0.8, and 1.0 wt%), and five formulations containing PLIL-modified black TiO_2_ at the same concentrations. Accordingly, a total of 33 independently fabricated disk specimens were produced for subsequent mechanical and tribological characterization.

### 2.4. Structural, Morphological, and Chemical Characterization

The morphology of untreated white and PLIL-modified black TiO_2_ nanoparticles was examined by field-emission scanning electron microscopy (FESEM) using an SU8020 microscope (Hitachi High-Technologies, Tokyo, Japan). The nanopowders were mounted on conductive carbon adhesive tape prior to observation. Secondary-electron images were acquired at an accelerating voltage of 2 kV, a beam current of 10 μA, and a working distance of approximately 12 mm. Micrographs were acquired at different magnifications, and at least five randomly selected and non-overlapping fields were examined for each nanoparticle condition.

The analysis focused on qualitative assessment of particle morphology, surface appearance, agglomeration, and PLIL-induced morphological changes. Because extensive particle agglomeration and overlap prevented reliable delineation of a statistically representative number of individual primary particles, quantitative particle-size distributions were not derived from the FESEM images. Accordingly, the FESEM analysis was restricted to qualitative morphological comparison, and the nominal particle-size range of 10–30 nm refers exclusively to the supplier specification for untreated white TiO_2_.

Accordingly, no experimentally determined mean primary-particle diameter or particle-size distribution is assigned to either nanoparticle condition. In particular, the supplier-reported 10–30 nm range for untreated TiO_2_ was not extrapolated to the PLIL-modified material. Complementary techniques capable of distinguishing primary-particle dimensions from agglomerate or hydrodynamic size would be required for a rigorous quantitative comparison of particle-size distributions before and after PLIL treatment.

The crystalline phases of untreated white and PLIL-modified black TiO_2_ nanoparticles were characterized by X-ray diffraction (XRD) using an Empyrean diffractometer (PANalytical, Almelo, The Netherlands) equipped with Co Kα radiation (λ = 0.1790 nm). Diffraction patterns were collected over a 2θ range of 20–80° at an operating voltage of 40 kV and a tube current of 40 mA, using a step size of 0.02° and a counting time of 1 s per step. Phase identification was performed by comparison with reference data from the International Centre for Diffraction Data database using anatase TiO_2_ (PDF 01-073-1764) and rutile TiO_2_ (PDF 04-004-4337).

Quantitative phase analysis was performed by Rietveld refinement using Materials Analysis Using Diffraction (MAUD, version 2.9998). The diffraction patterns of untreated white and PLIL-modified black TiO_2_ were modeled using anatase and rutile as the crystalline phases. The Rietveld method employs a least-squares refinement procedure in which the calculated diffraction profile is iteratively adjusted to minimize the weighted difference between the observed and calculated diffraction intensities. The relative weight fraction of each crystalline phase was determined from its refined scale factor according to *W_i_* = *S_i_*(*ZMV*)*_i_*/*∑_j_ S_j_*(*ZMV*)_j_, where *S_i_* is the refined scale factor, *Z* is the number of formula units per unit cell, *M* is the molecular weight of the formula unit, and *V* is the unit-cell volume. Refinement included optimization of the background, phase scale factors, zero-point shift, lattice parameters, preferred-orientation parameters, peak-width parameters, asymmetry, and peak-shape parameters. Peak profiles were modeled using a pseudo-Voigt function. The same refinement strategy and crystallographic models were applied to untreated white and PLIL-modified black TiO_2_ to ensure consistent quantitative comparison. Refinement was continued until convergence was achieved. The quality of the refinements was assessed by inspection of the agreement between the experimental and calculated diffraction profiles and by monitoring the weighted-profile residual (*R_wp_*) and goodness-of-fit (χ^2^) parameters. The resulting quantitative phase fractions and final refinement-quality indicators are reported in Section 3.2 because they constitute outputs of the Rietveld analysis rather than predefined refinement parameters.

The FESEM and XRD measurements were designed to compare the untreated white and PLIL-modified nanoparticle conditions and were not conducted as a batch-resolved quantitative reproducibility analysis of the independently irradiated PLIL preparations.

Raman spectroscopy was performed on untreated white and PLIL-modified black TiO_2_ nanoparticles using a DXR Raman microscope (Thermo Fisher Scientific, Waltham, MA, USA). Raman spectra were acquired using a 532 nm excitation laser and a 50× microscope objective. The laser power at the specimen was maintained at ≤2 mW to minimize local heating and potential laser-induced modification during acquisition. Spectra were collected over the range of 100–1000 cm^−1^ with a spectral resolution of ≤4 cm^−1^. For each measurement, three consecutive acquisitions of 10 s each were accumulated to improve the signal-to-noise ratio. Prior to spectral acquisition, the Raman spectrometer was calibrated using the characteristic Raman band of a silicon reference at approximately 520.7 cm^−1^.

Raman spectroscopy was used as a complementary technique to XRD for crystalline-phase identification and comparison of anatase- and rutile-associated spectral contributions following PLIL treatment. FESEM, XRD, and Raman spectroscopy were not used to determine Ti oxidation state, oxygen-vacancy concentration, or the chemical or electronic origin of the visible PLIL-induced darkening.

Fourier transform infrared spectroscopy (FTIR) was used to investigate the chemical structure of UHMWPE and determine whether hot pressing induced detectable oxidation. Spectra were acquired from 4000 to 400 cm^−1^ with a spectral resolution of 4 cm^−1^ using attenuated total reflectance (ATR) mode. The analyses were carried out using FTIR spectrometers (Nicolet iS50, Thermo Scientific, Waltham, MA, USA). Prior to analysis, all spectra were baseline corrected and normalized to facilitate direct comparison among samples.

SEM–EDS analysis was additionally performed to assess the spatial distribution of Ti-containing features within the UHMWPE matrix. The composite containing 1.0 wt% TiO_2_, corresponding to the highest nanoparticle concentration investigated, was selected for this analysis to evaluate filler distribution under the condition with the greatest expected susceptibility to localized particle accumulation within the studied composition range. Two spatially separated regions of the consolidated specimen were examined: a central region and a region near the specimen edge. SEM imaging and EDS acquisition were performed at an accelerating voltage of 20 kV using an energy-dispersive X-ray detector with an energy resolution of approximately 127 eV. The central region was analyzed at 2000× magnification, whereas the edge region was examined at 20,000× magnification to provide complementary information at a higher spatial magnification. For each region, the spatial distributions of C, O, and Ti were evaluated using C/O/Ti elemental overlays and Ti Kα elemental maps, together with the corresponding EDS sum spectra. Local elemental compositions were estimated from the sum spectra using the instrument software and are reported as weight percentages. Because EDS probes a localized electron–matter interaction volume, these measurements were used to assess the spatial occurrence of Ti-containing features within the carbon-rich UHMWPE matrix and were not interpreted as bulk quantitative determinations of the nominal TiO_2_ loading.

Following tribological testing, representative wear tracks were examined by SEM using standardized imaging conditions. At least five representative regions per selected wear track were evaluated to avoid interpretation from a single field of view.

Post-tribological chemical characterization of the polymer wear tracks, generated wear debris, and H13 steel counterbody surfaces by EDS or FTIR was not performed in the present study. Accordingly, the post-sliding SEM examination was used to assess worn-surface morphology rather than to determine chemical changes or material transfer across the tribological interface. No direct before/after compositional comparison of the specimen and counterbody surfaces is therefore inferred from these measurements.

### 2.5. Vickers Microhardness and Instrumented Indentation Load–Displacement Response

Vickers microhardness was measured using an HMV-2 digital microhardness tester (Shimadzu Corporation, Kyoto, Japan) in accordance with ASTM E384 [33]. A test force of 490 mN (approximately 50 gf; HV0.05) was applied for 15 s. Three independently fabricated disks were evaluated for each formulation (*n* = 3 independent specimens), with five Vickers indentations performed at different locations on each disk.

The five measurements obtained from each disk were averaged to yield one specimen-level microhardness value. These specimen-level means were treated as the independent observations for statistical analysis (*n* = 3 per formulation). Individual indentations were separated by a distance of at least three times the mean indentation diagonal and positioned sufficiently far from specimen edges to minimize interaction between adjacent deformation zones and edge effects. Microhardness values are reported as mean ± standard deviation (*SD*) calculated from the three independent specimen-level means.

Instrumented indentation measurements were performed using an SMT-2000 PiQ instrumented indenter (Rtec Instruments, San Jose, CA, USA) equipped with a Berkovich diamond tip. A maximum load of 200 mN was applied, followed by a 10 s holding period at maximum load and a 10 s unloading segment. The applied load (*P*) and penetration depth (*h*) were continuously recorded during the loading–holding–unloading cycle to obtain load–displacement curves.

Three independently fabricated disks were examined for each formulation, and five indentations were acquired at different locations on each disk. In the present study, the instrumented-indentation measurements were used exclusively as a descriptive and comparative assessment of local load–penetration behavior rather than for quantitative determination of indentation hardness or reduced elastic modulus. Accordingly, indentation hardness and modulus values were not calculated, and the instrumented-indentation measurements were not included in inferential statistical analyses. Representative load–displacement curves were selected to illustrate qualitative variations in local indentation behavior among the investigated formulations.

Because UHMWPE exhibits pronounced time-dependent and viscoelastic deformation and because the present measurements were not designed for validated quantitative extraction of modulus or indentation hardness, interpretation was restricted to the directly recorded load–displacement behavior and approximate penetration depths observed in representative curves. The instrumented-indentation results were therefore considered complementary to, rather than equivalent to, the quantitative Vickers microhardness measurements.

### 2.6. Dry Ball-on-Disc Tribological Testing

Dry sliding tests were conducted using a laboratory-developed ball-on-disc tribometer (UANL, Monterrey, Nuevo León, Mexico) in accordance with ASTM G99 [34]. The tribometer was equipped with a load cell for continuous measurement of tangential friction force and an integrated optical imaging system for monitoring the wear track. Disk specimens had nominal dimensions of 30 mm in diameter and 4 mm in thickness.

Prior to tribological testing, specimen surfaces were finished using 1000-grit SiC abrasive paper following a standardized surface-finishing procedure. The finishing direction, applied load, and treatment duration were maintained constant for all specimens. After surface preparation, the specimens were cleaned with ethanol to remove residual abrasive particles and surface contaminants and subsequently dried at room temperature before tribological testing.

The arithmetic mean surface roughness (*R_a_*) and areal surface roughness (*S_a_*) of the polymer specimens were not independently quantified by contact or optical profilometry before tribological testing. Therefore, no numerical roughness value is assigned retrospectively from the abrasive-paper grade. The standardized 1000-grit SiC finishing procedure was applied identically to all specimens to establish a consistent nominal initial surface condition for comparative tribological testing; however, the absence of direct pre-test profilometric characterization should be considered when interpreting the absolute friction and wear response.

An 11 mm diameter H13 tool-steel ball (voestalpine High Performance Metals S.A. de C.V., Querétaro, Mexico) was used as the stationary counterbody. The hardness and initial surface roughness of the counterbody were 50–54 HRC and *R_a_* ≤ 0.05 μm, respectively. A new ball or an unused region of the ball surface was used for each independent tribological test to prevent pre-existing wear damage from influencing the measurements.

Tests were performed under a normal load of 30 N at a rotational speed of 250 rpm, corresponding to an angular velocity of approximately 26.18 rad s^−1^. At a wear-track radius of 7.5 mm, the corresponding nominal linear sliding velocity was approximately 0.196 m s^−1^. The total sliding distance was 530 m, corresponding to a test duration of approximately 45 min. All tribological tests were conducted under dry sliding conditions at an ambient temperature of 23 ± 2 °C and a relative humidity of 50 ± 10%.

The tangential friction force was continuously measured using the tribometer load cell, and the instantaneous coefficient of friction (COF), μ, was calculated as:

(1) $$µ=\;\frac{F_{t}}{F_{N}}$$

where *F_t_* is the measured tangential friction force (*N*) and *F_N_* is the applied normal load (30 N).

The complete coefficient-of-friction curves were evaluated as a function of sliding time and distance to examine friction evolution during testing. Inspection of the friction curves revealed an initial running-in period followed by gradual and formulation-dependent evolution of the friction response. Therefore, a strictly invariant steady-state regime common to all formulations could not be unambiguously identified.

To provide an objective and consistent quantitative comparison, the mean terminal coefficient of friction, μ_terminal_, was calculated over the final 400–530 m of sliding distance, corresponding to approximately 2040–2700 s at a nominal sliding velocity of 0.196 m s^−1^:

(2) $${\bar{µ}}_{\mathrm{terminal}}=\frac{1}{N}\sum_{i=1}^{N}{µ}_{i}$$

where μ_i_ is the instantaneous coefficient of friction at the *i*th data point and *N* is the total number of data points recorded within the selected terminal interval.

The same 400–530 m interval was applied to all specimens and formulations. This interval was employed solely as a standardized comparison window and was not assumed to represent a universal steady-state friction regime.

Three independently fabricated specimens were tested for each formulation (*n* = 3), with one independent ball-on-disc test performed per specimen. The resulting specimen-level mean terminal coefficients of friction were reported as mean ± *SD* calculated from the three independent tests.

### 2.7. Wear-Volume Determination and Worn-Surface Analysis

Following each tribological test, wear-track width was measured using a calibrated Supereyes B008 digital USB microscope (5.0 MP; Shenzhen Supereyes Technology Co., Ltd., Shenzhen, China) coupled with Supereyes measurement software (version 3.5.3). Prior to wear-track measurements, the imaging system was spatially calibrated using a reference scale at the same magnification employed for image acquisition.

The nominal 5.0-MP pixel count of the digital microscope should not be interpreted as the dimensional sensitivity of the wear-track measurement. The effective spatial sampling and dimensional measurement capability depend on the acquisition magnification, field of view, and pixel-to-length conversion established by spatial calibration. Because an independently validated dimensional uncertainty or minimum detectable track-width increment was not established for the complete microscope–software measurement chain, a specific micrometer-scale instrumental sensitivity is not assigned here. Accordingly, the numerical precision used for the derived wear quantities was selected to avoid implying accuracy beyond that supported by the calibrated width-based measurement procedure: wear volume is reported to two decimal places in mm^3^, and specific wear rate is reported to two decimal places in the coefficient expressed as ×10^−4^ mm^3^ N^−1^ m^−1^.

To reduce the influence of local circumferential variations in wear-track geometry, the track width was measured at eight approximately equally spaced positions around each wear track. These eight measurements represented technical measurements of the same specimen and were not treated as statistically independent observations. The mean wear-track width obtained from the eight circumferential positions was used to calculate one specimen-level wear-volume estimate for each independently tested disk.

Wear volume was estimated using a geometrical approximation in which the mean cross-section of the groove generated by the spherical counterbody was represented as a circular segment. This calculation assumes that the mean geometrical relationship between wear-track width and spherical-counterbody curvature is representative of the circumference of the wear track. The estimated cross-sectional area of the wear groove, *A*, was calculated according to:

(3) A=r2sin−1d/2r−d/44r2−d212

where *A* is the estimated cross-sectional area of the wear groove (mm^2^), *r* is the radius of the spherical counterbody (5.5 mm), and *d* is the mean wear-track width (mm) obtained from the eight circumferential measurements. The inverse-sine term was evaluated in radians.

The corresponding estimated volumetric wear loss, *V*, was calculated by multiplying the estimated mean cross-sectional area by the circumference of the circular wear track:

*V* = 2*πRA* (4)

where *V* is the estimated volumetric wear loss (mm^3^), *R* is the wear-track radius (7.5 mm), and *A* is the estimated mean cross-sectional wear-groove area (mm^2^). The specific wear rate, *k*, was subsequently calculated as:

(5) $$k=\frac{V}{F_{N}L}$$

where *V* is the estimated wear volume, *F_N_* is the applied normal load (30 N), and *L* is the total sliding distance (530 m). The specific wear rate is reported in mm^3^ N^−1^ m^−1^. This geometric approximation is widely adopted for ball-on-disc tests, and systematic errors may originate from polymer pile-up and viscoelastic recovery of UHMWPE.

The width-based circular-segment procedure constitutes an idealized geometrical approximation rather than a direct three-dimensional measurement of material loss. For UHMWPE, plastic deformation, material pile-up, time-dependent viscoelastic recovery, and local irregularities in wear-groove morphology may cause the actual cross-section to deviate from the ideal spherical-segment geometry assumed in Equation (3). The use of eight circumferential width measurements reduces the influence of local width variations but does not eliminate systematic uncertainty associated with the assumed cross-sectional geometry.

Independent validation of the calculated wear volumes by three-dimensional optical profilometry or stylus profilometry was not performed in the present study. Consequently, the reported wear-volume values should be regarded as standardized geometrical estimates obtained using the same procedure for all specimens. Small differences among formulations were therefore interpreted in conjunction with specimen-to-specimen variability and the corresponding inferential statistical analysis rather than solely from the numerical ranking of wear-volume estimates.

Wear of the H13 steel counterbody was not included in the volumetric-loss calculation. Following testing, selected worn surfaces were examined by SEM as described in Section 2.4.

### 2.8. Statistical Analysis

Quantitative data were reported as mean ± standard deviation (*SD*), with independent experimental specimens clearly distinguished from repeated technical measurements. Three independently fabricated disks were evaluated for each material condition (*n* = 3). Repeated measurements performed on the same disk were considered technical measurements and were not treated as statistically independent observations.

For Vickers microhardness, the five technical measurements obtained from each independently fabricated disk were averaged to obtain one specimen-level microhardness value. Accordingly, the independently fabricated disk, rather than each individual indentation, was considered the experimental unit for statistical analysis, resulting in three independent specimen-level values per formulation (*n* = 3).

Instrumented-indentation load–displacement measurements were evaluated descriptively and were not included in inferential statistical analyses. Individual instrumented-indentation curves were therefore treated as local technical measurements used to examine qualitative variations in load–penetration behavior rather than as statistically independent observations or quantitative formulation-level mechanical properties.

For the tribological measurements, one independent ball-on-disc test was performed on each of the three independently fabricated disks per material condition. Consequently, volumetric wear loss and the mean terminal coefficient of friction calculated for each test were treated as independent specimen-level observations, resulting in *n* = 3 independent values per formulation. Friction data points continuously acquired during an individual ball-on-disc test were not treated as independent statistical replicates.

Specific wear rate was calculated directly from the specimen-level volumetric wear loss using the same constant normal load and sliding distance for all tests. Because specific wear rate therefore represents a constant positive linear transformation of volumetric wear loss, it was not subjected to a separate inferential statistical analysis; such an analysis would yield the same relative statistical conclusions as those obtained for volumetric wear loss.

For the TiO_2_-reinforced formulations, a two-way analysis of variance (ANOVA) was used to evaluate the main effects of nanoparticle state, defined for the statistical model as untreated white TiO_2_ versus PLIL-modified black TiO_2_, TiO_2_ concentration (0.1, 0.3, 0.5, 0.8, and 1.0 wt%), and their interaction (nanoparticle state × concentration). Neat UHMWPE was excluded from the two-way factorial ANOVA because nanoparticle state is not defined for the unreinforced control. Comparisons between individual TiO_2_-containing formulations and neat UHMWPE were therefore performed separately using Dunnett-adjusted multiple comparisons against the neat-UHMWPE control.

Before interpretation of the parametric analyses, the assumptions of the ANOVA models were evaluated from the model residuals. Residual normality was assessed primarily by inspection of normal Q–Q plots and complemented by the Shapiro–Wilk test, whereas homogeneity of variance was evaluated using Levene’s test. Because the number of independent specimens per formulation was limited (*n* = 3), formal normality tests were interpreted cautiously and were not used as the sole criterion for determining the suitability of the parametric analyses.

Inspection of the residual diagnostics did not identify substantial departures from residual normality or homogeneity of variance that required data transformation or replacement of the planned parametric analyses. Accordingly, all ANOVA models were applied to the untransformed specimen-level data.

Statistical significance was established at *α* = 0.05. Exact *p*-values were reported whenever appropriate. For the factorial ANOVA, partial *η*^2^ was reported as an estimate of effect size. For Dunnett-adjusted comparisons against neat UHMWPE, the estimated difference (Δ) relative to the control and the corresponding multiplicity-adjusted *p*-value were reported. Because 95% confidence intervals were not calculated and reported consistently for all factorial effects and Dunnett-adjusted contrasts, no systematic reporting of 95% confidence intervals is claimed. Statistical analyses were performed using OriginPro 2025 (OriginLab Corporation, Northampton, MA, USA).

Three independent specimens (*n* = 3) were evaluated per material condition. The relatively small sample size may limit the statistical power to detect moderate differences between formulations. Consequently, non-significant results should not be interpreted as definitive evidence of equivalent mechanical or tribological performance, and the observed numerical differences should be considered in conjunction with specimen-to-specimen variability and the corresponding statistical analyses.

## 3. Results

### 3.1. Morphology of White and PLIL-Modified Black TiO_2_ Nanoparticles

**Figure 1:**
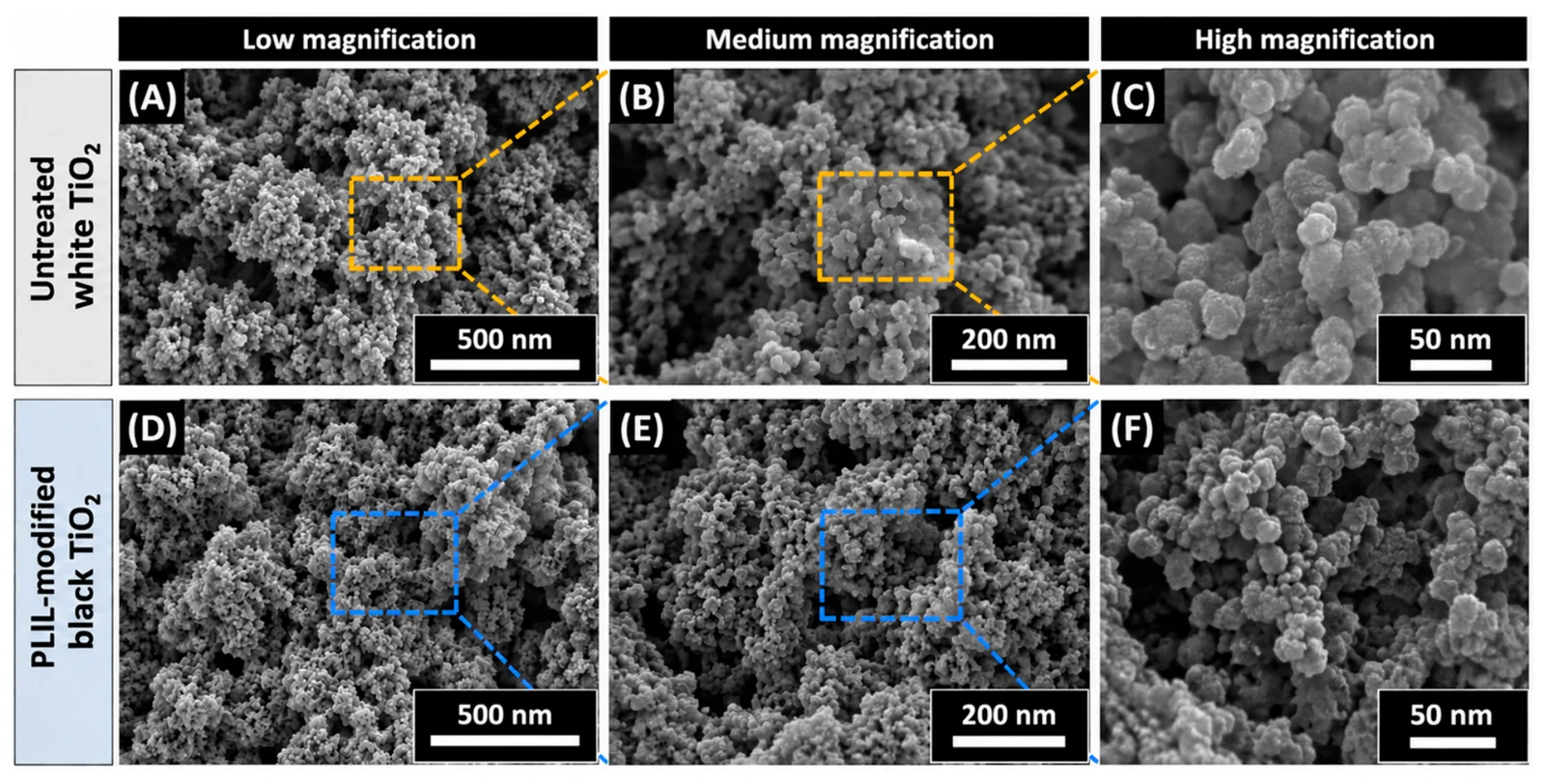
Representative FESEM micrographs of untreated white TiO_2_ nanoparticles (**A**–**C**) and PLIL-modified black TiO_2_ nanoparticles (**D**–**F**) acquired at progressively higher magnifications. Panels (**A**,**D**) show the lower-magnification morphology, panels (**B**,**E**) provide intermediate-magnification views, and panels (**C**,**F**) show higher-magnification morphological details. Scale bars are indicated in each micrograph. Note: FESEM micrographs are used only for qualitative morphological comparison. Quantitative particle-size distribution analysis was not performed due to extensive particle agglomeration.

Figure 1 compares representative FESEM micrographs of untreated white TiO_2_ and PLIL-modified black TiO_2_ nanopowders acquired at different magnifications. The untreated white TiO_2_ consisted predominantly of irregular particles with angular and faceted morphologies, together with a smaller fraction of rounded particles. Agglomerated regions composed of finer particles were also observed. In contrast, the PLIL-modified black TiO_2_ exhibited a markedly greater prevalence of rounded and nearly spherical morphologies. Larger spherical features associated with agglomerated regions were additionally evident after laser irradiation.

These observations establish a clear qualitative morphological difference between the untreated and PLIL-modified TiO_2_ powders. The recovered PLIL-treated powder also exhibited a visibly darker appearance than the untreated white TiO_2_ precursor.

These FESEM observations establish a qualitative PLIL-induced morphological transformation but are not used to infer a quantitative change in primary-particle size because individual particle boundaries could not be resolved across a sufficiently large, statistically representative population.

### 3.2. X-Ray Diffraction and Phase Composition

**Figure 2:**
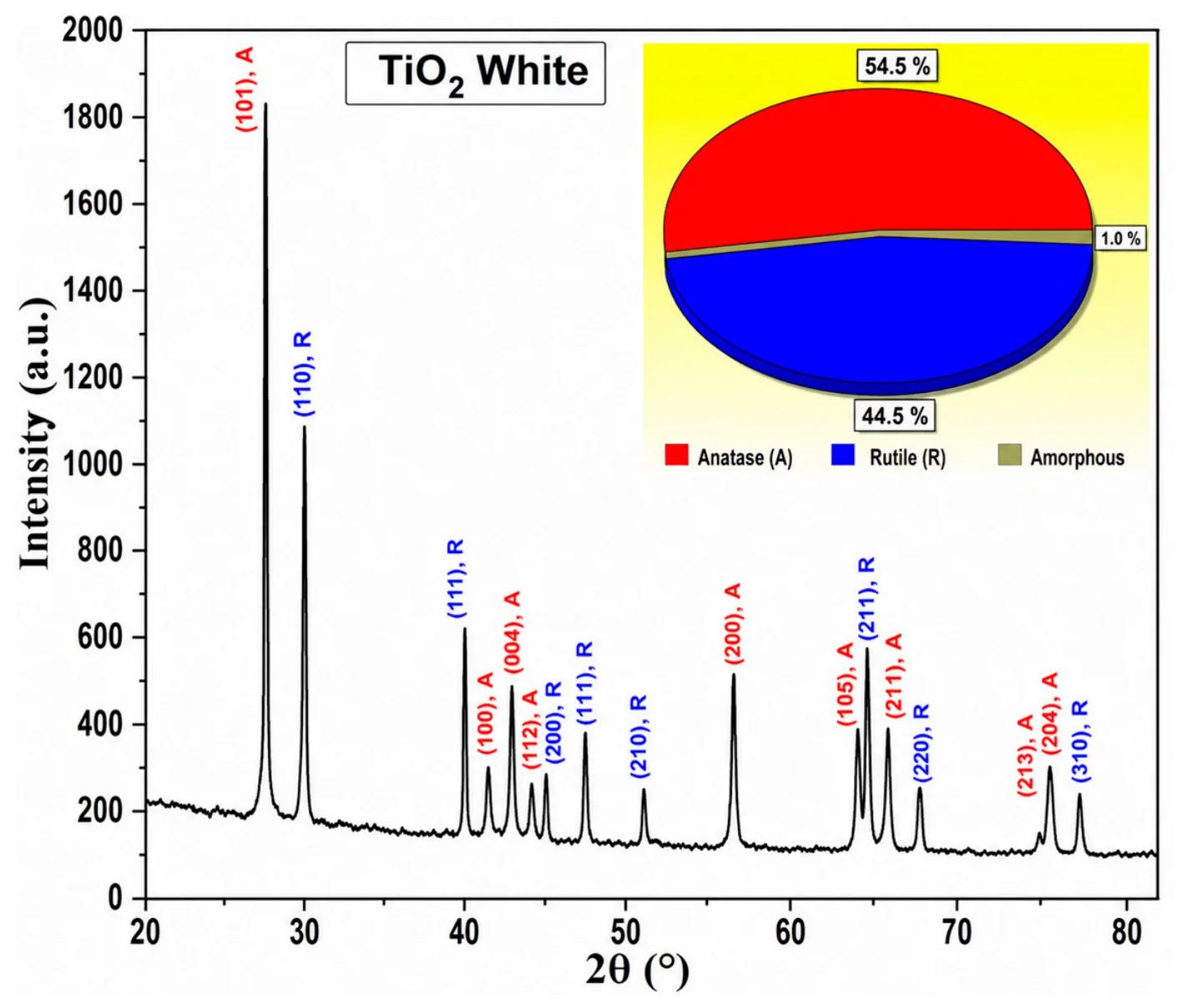
X-ray diffraction pattern of untreated white TiO_2_ nanoparticles. A and R indicate anatase and rutile, respectively.

**Figure 3:**
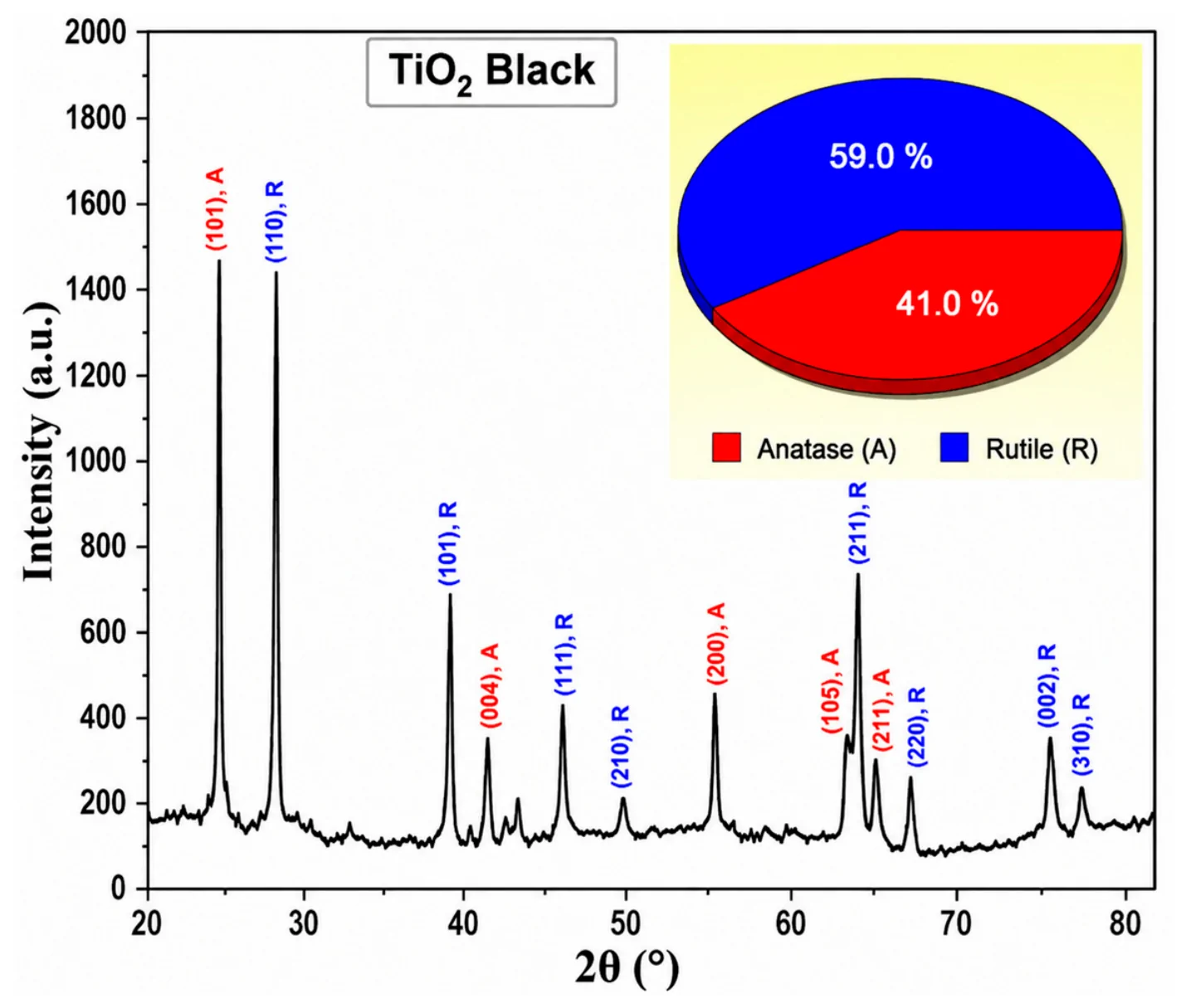
X-ray diffraction pattern of PLIL-modified black TiO_2_ nanoparticles. A and R indicate anatase and rutile, respectively.

Figure 2 and Figure 3 show the XRD patterns of untreated white TiO_2_ and PLIL-modified black TiO_2_, respectively. Both nanopowders contained anatase and rutile crystalline phases. For untreated white TiO_2_, the diffraction peaks were indexed using anatase PDF 01-073-1764 and rutile PDF 04-004-4337. The pattern was anatase-dominant, with Rietveld quantitative phase analysis yielding approximately 56 wt% anatase and 44 wt% rutile.

Following PLIL treatment, reflections corresponding to both TiO_2_ polymorphs remained present and were indexed using anatase PDF 01-073-1764 and rutile PDF 04-004-4337. However, the relative contribution of the rutile reflections increased. Quantitative crystalline-phase analysis yielded approximately 41 wt% anatase and 59 wt% rutile, normalized to the total crystalline-phase content, indicating a shift from an anatase-dominant phase assemblage in untreated white TiO_2_ to a rutile-dominant crystalline-phase balance in PLIL-modified black TiO_2_. Relative to the untreated powder, this corresponded to an approximately 15-percentage-point increase in the refined rutile fraction, accompanied by a corresponding decrease in the anatase fraction.

The Rietveld refinement of untreated white TiO_2_ yielded a weighted-profile residual (*R_wp_*) of 7.10% and a goodness-of-fit (χ^2^) of 2.42, whereas the PLIL-modified black TiO_2_ yielded *R_wp_* = 6.17% and χ^2^ = 2.21. The weighted-profile residual (*R_wp_*) quantifies the weighted discrepancy between the observed and calculated diffraction profiles, whereas χ^2^ provides a goodness-of-fit measure. Although *R_wp_* values below 10% are frequently regarded as indicative of favorable agreement in suitable powder-diffraction datasets, no universal acceptance threshold applies to all Rietveld refinements. These refinement-quality indicators, together with the agreement between the calculated and experimental diffraction profiles, support the adequacy of the anatase–rutile structural models used for quantitative phase analysis. Thus, PLIL treatment preserved the coexistence of the two TiO_2_ polymorphs while producing a measurable redistribution of their relative crystalline-phase fractions toward a rutile-dominant composition. Previous investigations have shown that the anatase-to-rutile transformation in black TiO_2_ can be influenced by structural defects and oxygen-vacancy-related phenomena [35]. Nevertheless, because the present study did not include defect-sensitive characterization, the observed increase in rutile fraction following PLIL treatment cannot be attributed specifically to oxygen vacancies or any other particular defect-mediated transformation mechanism.

The Miller indices and phase assignments of the principal diffraction reflections are indicated in Figure 2 and Figure 3. The Rietveld-refinement methodology used to determine the anatase and rutile weight fractions and to assess refinement quality is described in Section 2.4.

### 3.3. Raman Spectroscopy

**Figure 4:**
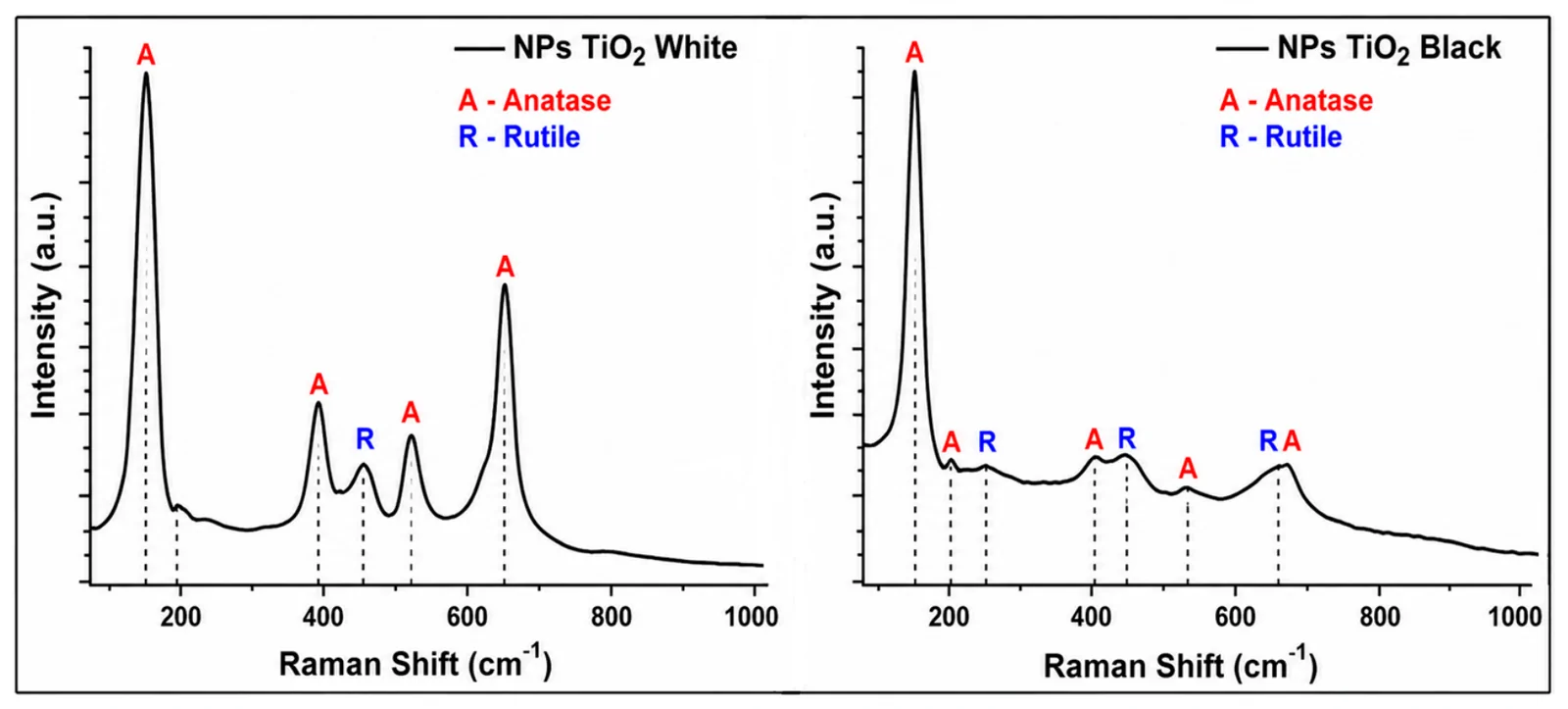
Raman spectra of untreated white TiO_2_ and PLIL-modified black TiO_2_ nanoparticles. The principal anatase- and rutile-related bands are indicated.

Figure 4 compares the Raman spectra of untreated white TiO_2_ and PLIL-modified black TiO_2_. The untreated white TiO_2_ spectrum exhibited bands centered at approximately 143, 196, 395, 445, 515, and 635 cm^−1^. The bands at approximately 143, 196, 395, 515, and 635 cm^−1^ were predominantly associated with anatase-related vibrational modes, whereas the contribution near 445 cm^−1^ was associated with rutile.

The PLIL-modified black TiO_2_ spectrum exhibited bands at approximately 143, 196, 235, 395, 445, 515, 612, and 632 cm^−1^. In addition to the anatase-related contributions, the bands near 445 and 612 cm^−1^ were consistent with an increased rutile contribution. The Raman spectra therefore corroborated the coexistence of anatase and rutile and the redistribution toward a larger rutile fraction observed by XRD.

Taken together, FESEM, XRD, and Raman spectroscopy demonstrate PLIL-induced morphological changes and redistribution of the anatase/rutile crystalline-phase balance, while the recovered PLIL-treated powder exhibited a visibly darker appearance than the untreated precursor. However, these measurements do not establish the chemical or electronic origin of the dark coloration. Accordingly, the present results do not provide direct evidence of Ti^3+^ formation, oxygen vacancies, TiO_2_ reduction, surface disorder, or a specific defect-related mechanism responsible for the observed blackening.

### 3.4. FTIR Analysis of the UHMWPE Nanocomposites

**Figure 5:**
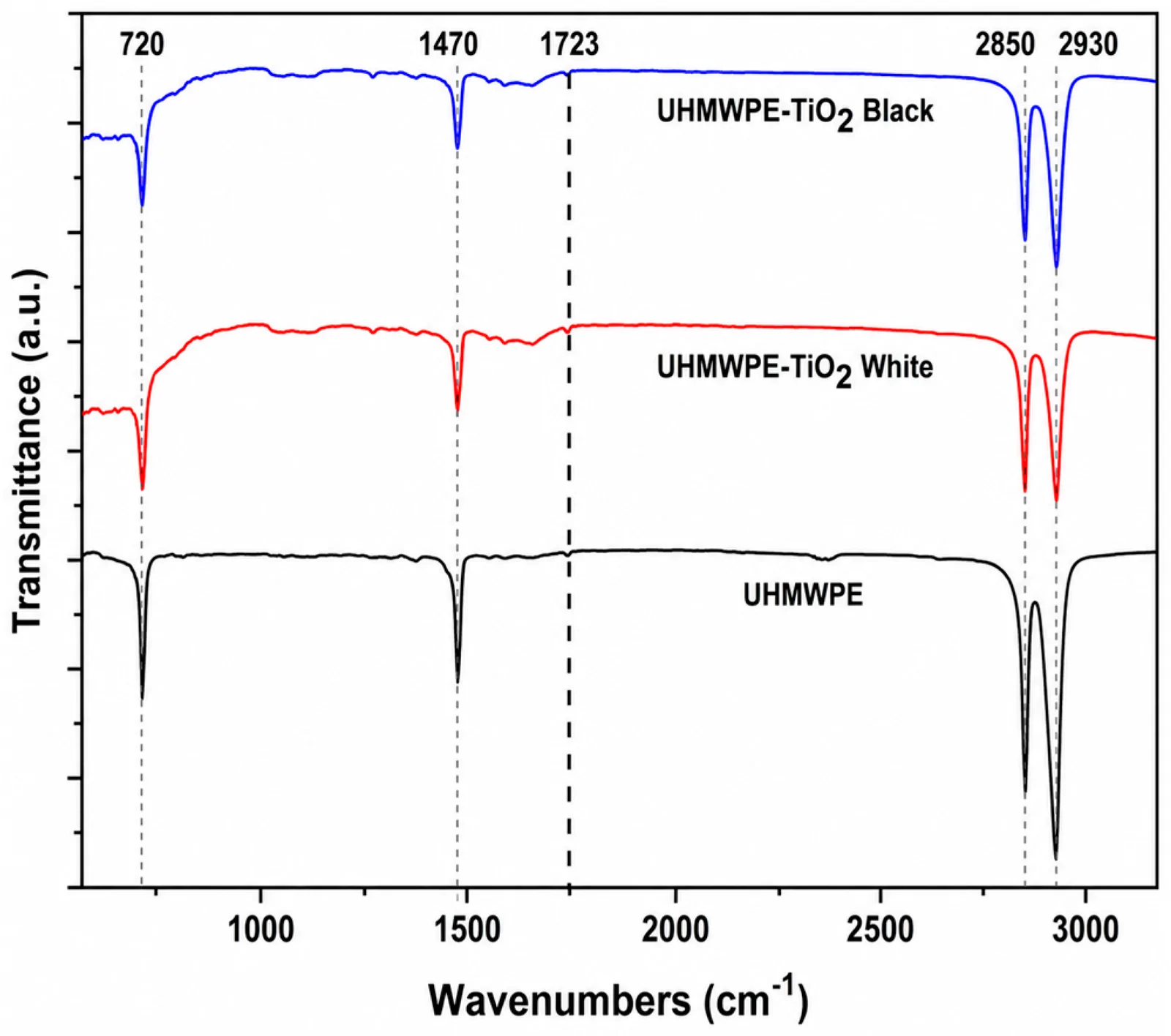
FTIR spectra of neat UHMWPE and representative UHMWPE nanocomposites containing untreated white and PLIL-modified black TiO_2_ after hot pressing.

Figure 5 shows the FTIR spectra of neat UHMWPE and representative nanocomposites containing untreated white and PLIL-modified black TiO_2_ after hot pressing at 180 °C for 15 min. The characteristic absorption bands of UHMWPE were retained after processing.

No detectable absorption feature associated with carbonyl-containing oxidation products was observed in the approximately 1720–1730 cm^−1^ region for the analyzed specimens. Within the detection capability of the FTIR measurements, the selected hot-pressing conditions therefore did not produce measurable oxidative modification of the UHMWPE matrix.

### 3.5. EDS Elemental Mapping and SPATIAL Distribution of TiO_2_ Within the UHMWPE Matrix

**Figure 6:**
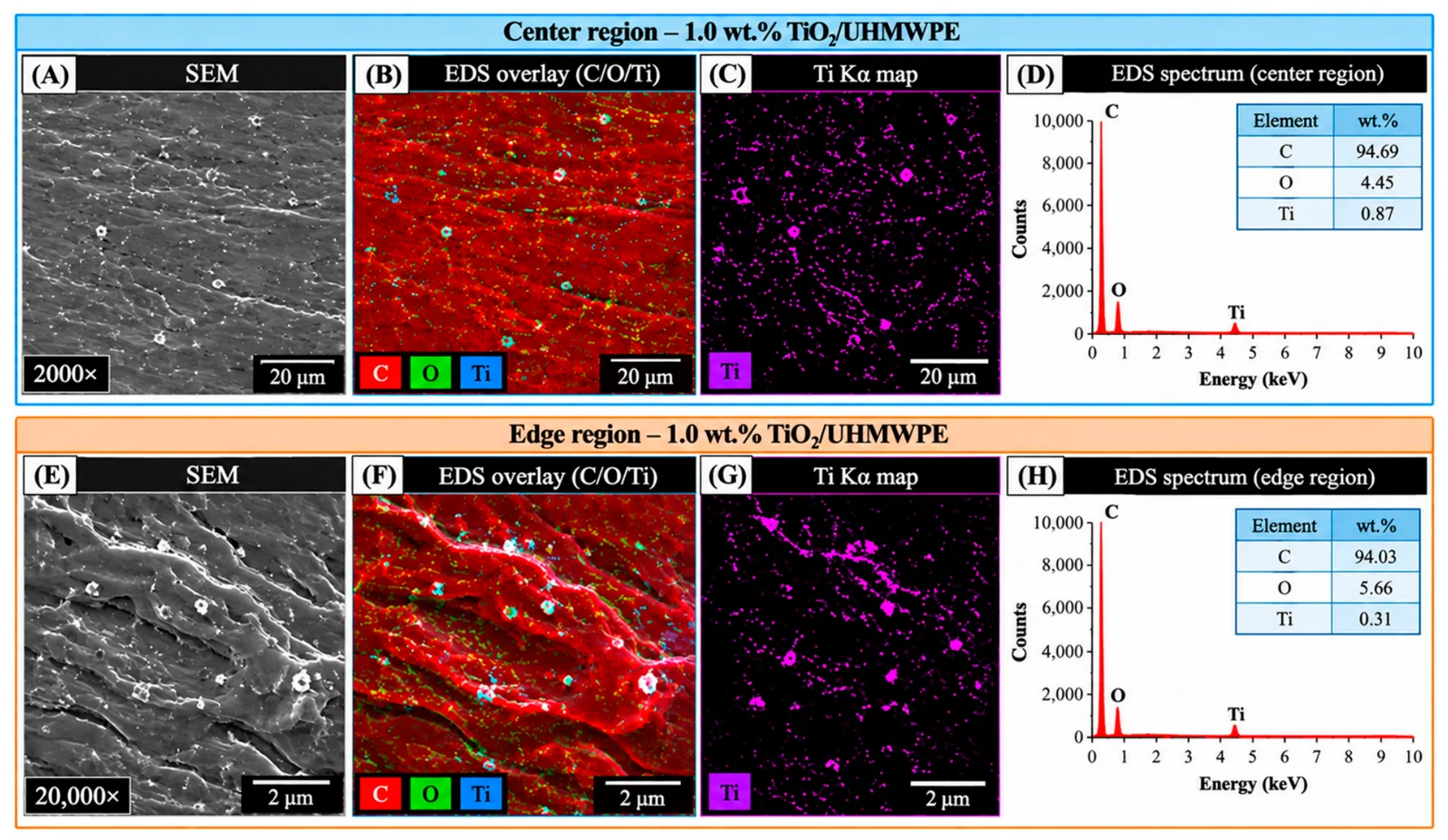
SEM–EDS assessment of TiO_2_ spatial distribution in the UHMWPE nanocomposite containing 1.0 wt% TiO_2_ at two spatially separated specimen regions: (**A**) SEM image of the central region; (**B**) C/O/Ti elemental overlay at the center; (**C**) Ti Kα elemental map at the center; (**D**) corresponding EDS sum spectrum; (**E**) SEM image of the edge region; (**F**) C/O/Ti elemental overlay at the edge; (**G**) Ti Kα elemental map at the edge; and (**H**) corresponding EDS sum spectrum.

To complement the morphological characterization and directly assess the spatial distribution of the ceramic reinforcement within the UHMWPE matrix, EDS elemental mapping was performed on the composite containing 1.0 wt% TiO_2_, corresponding to the highest nanoparticle concentration investigated in this study. This formulation was selected as the most demanding condition for evaluating filler distribution because increasing nanoparticle content decreases the average interparticle separation and can increase the probability of particle–particle interactions and localized accumulation. Two spatially separated regions of the consolidated specimen were therefore examined: the central region and a region near the specimen edge (Figure 6).

In the central region (Figure 6A–D), the C/O/Ti elemental overlay and Ti Kα map reveal Ti-containing signals distributed throughout the analyzed field within the carbon-rich UHMWPE matrix. The corresponding sum-spectrum analysis detected C, O, and Ti, with measured concentrations of 94.69, 4.45, and 0.87 wt%, respectively. Examination of the specimen edge at higher magnification (Figure 6E–H) likewise revealed spatially distributed Ti signals, with the corresponding sum spectrum yielding 94.03 wt% C, 5.66 wt% O, and 0.31 wt% Ti. Thus, Ti was independently detected in two spatially distinct regions of the consolidated specimen.

The elemental maps do not show, within the analyzed fields, localization of the Ti signal into a single large Ti-rich domain; instead, Ti-containing features are distributed across both the central and edge regions. This observation is particularly relevant because it was obtained for the 1.0 wt% formulation, which represents the maximum TiO_2_ loading investigated and therefore the condition with the greatest expected susceptibility to localized particle accumulation within the present composition range. The EDS results consequently provide direct experimental evidence that the adopted dispersion, solvent-assisted mixing, and hot-pressing procedure produced spatial distribution of the Ti-containing phase across separated regions of the UHMWPE composite.

The local EDS concentrations should not, however, be interpreted as bulk measurements of the nominal TiO_2_ loading. The nominal formulation is expressed as TiO_2_ mass fraction, whereas EDS reports the elemental composition of a localized electron–matter interaction volume. In addition, the quantitative response is influenced by the analyzed area, interaction depth, local nanoparticle population, accelerating voltage, and the low-Z polymer matrix. The difference between the Ti contents measured in the center and edge regions therefore indicates local sampling variability and differences in the analyzed interaction volumes rather than, by itself, evidence of macroscopic filler segregation.

These results also refine the interpretation of the tribological behavior. The observation of distributed Ti signals at the maximum investigated filler loading makes gross micron-scale segregation within the analyzed regions an insufficient explanation, by itself, for the non-monotonic wear response. Nevertheless, the EDS maps do not establish perfectly homogeneous nanoparticle dispersion and cannot exclude sub-resolution TiO_2_ clusters, local particle-rich regions outside the analyzed fields, or concentration gradients at length scales below the spatial resolution of the measurement. Likewise, they do not directly demonstrate particle pull-out, third-body abrasion, or transfer-film formation during sliding. Consequently, the wear mechanisms discussed herein are restricted to interpretations consistent with the combined wear-volume, friction, worn-surface SEM, and EDS evidence rather than being assigned solely from morphological observations.

### 3.6. Mechanical Response

#### 3.6.1. Vickers Microhardness

**Figure 7:**
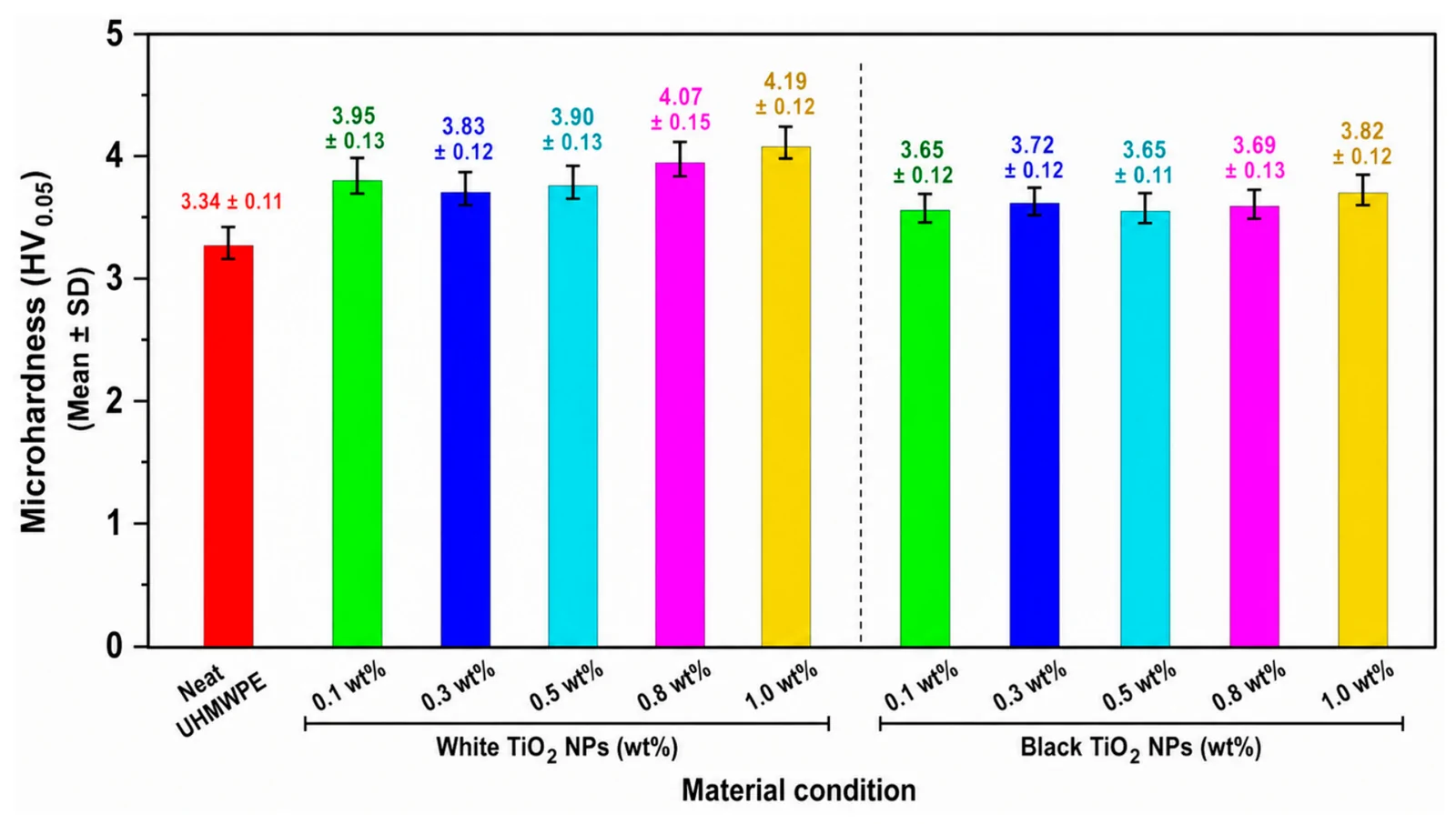
Vickers microhardness (HV0.05) of neat UHMWPE and UHMWPE nanocomposites reinforced with untreated white and PLIL-modified black TiO_2_. Values are reported as mean ± standard deviation (*SD*) from three independently fabricated specimens per formulation (*n* = 3).

Figure 7 summarizes the Vickers microhardness of neat UHMWPE and the nanocomposites containing untreated white and PLIL-modified black TiO_2_. Vickers microhardness measurements showed concentration-dependent differences between the untreated white-TiO_2_ and PLIL-modified black-TiO_2_ nanocomposites. Neat UHMWPE exhibited a mean microhardness of 3.34 ± 0.11 HV0.05. Incorporation of untreated white TiO_2_ increased the mean microhardness across the investigated concentration range, with the highest values obtained at 0.8 and 1.0 wt% TiO_2_, reaching 4.07 ± 0.15 and 4.19 ± 0.12 HV0.05, respectively. The latter corresponds to an increase of approximately 25% relative to neat UHMWPE.

The PLIL-modified black-TiO_2_ nanocomposites also exhibited higher mean Vickers microhardness values than neat UHMWPE, although their values were generally lower than those of the corresponding untreated white-TiO_2_ formulations. Among the PLIL-modified black-TiO_2_ composites, the highest mean microhardness was observed at 0.5 wt% TiO_2_, reaching approximately 3.82 ± 0.10 HV0.05. The microhardness response did not increase monotonically with TiO_2_ concentration, indicating that the mechanical response depended on both nanoparticle condition and filler concentration.

Although the absolute Vickers microhardness values remained within a relatively narrow range, consistent with the low TiO_2_ contents investigated (0.1–1.0 wt%), their numerical proximity should not be interpreted as statistical equivalence or as evidence of an absence of filler influence. Specimen-level inferential analysis demonstrated statistically significant main effects of both nanoparticle state and TiO_2_ concentration on Vickers microhardness. Moreover, Dunnett-adjusted comparisons against neat UHMWPE showed that all investigated TiO_2_-containing formulations exhibited significantly higher Vickers microhardness than the unreinforced control (*p* < 0.05). Accordingly, within the investigated low-filler regime, TiO_2_ incorporation produced a statistically supported but moderate and formulation-dependent increase in Vickers microhardness. The corresponding inferential statistical analysis is presented in Section 3.8.1.

#### 3.6.2. Instrumented Indentation Load–Displacement Response

**Figure 8:**
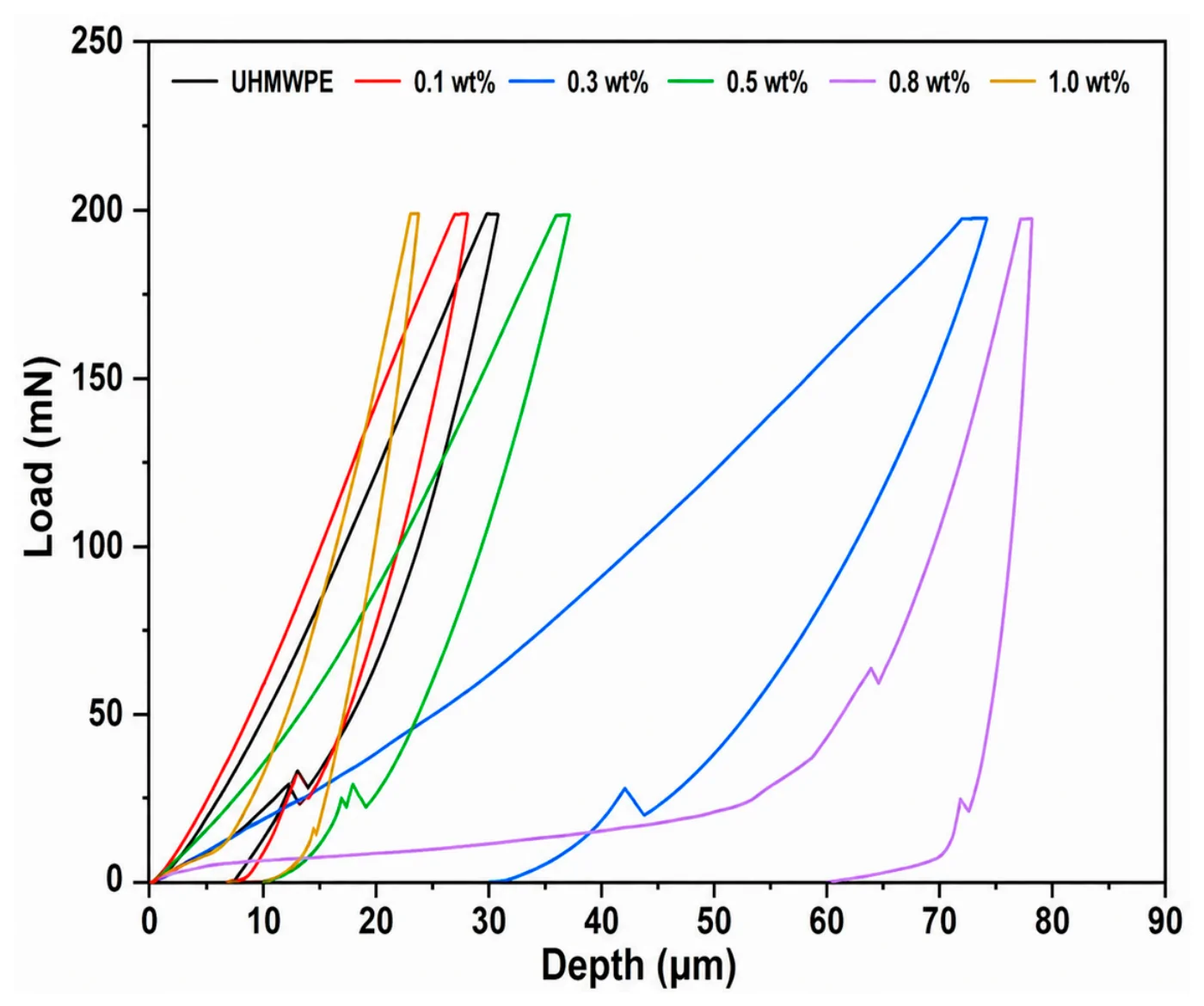
Representative instrumented-indentation load–displacement curves for neat UHMWPE and UHMWPE nanocomposites containing untreated white TiO_2_. The curves are presented to illustrate the local load–penetration response under a maximum applied load of 200 mN and are not intended to represent formulation-level mean indentation properties.

**Figure 9:**
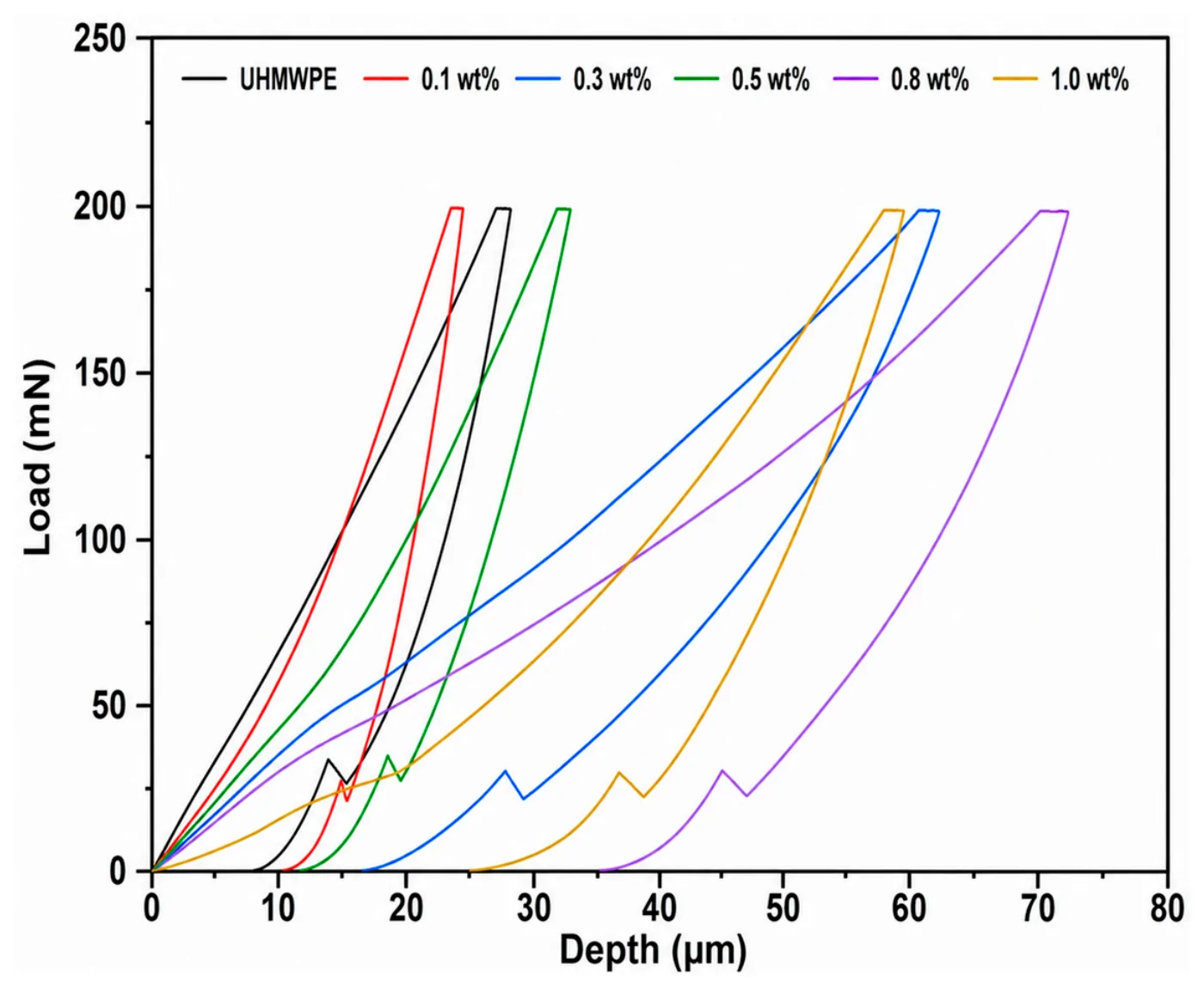
Representative instrumented-indentation load–displacement curves for neat UHMWPE and UHMWPE nanocomposites containing PLIL-modified black TiO_2_. The curves are presented to illustrate the local load–penetration response under a maximum applied load of 200 mN and are not intended to represent formulation-level mean indentation properties.

All presented load–displacement curves correspond to local indentation events and do not represent bulk-level mechanical properties for each composite formulation. Figure 8 and Figure 9 present representative instrumented-indentation load–displacement curves obtained for neat UHMWPE and the nanocomposites containing untreated white and PLIL-modified black TiO_2_, respectively, under a maximum applied load of 200 mN. These measurements were used to compare the local load–penetration behavior of the materials and were not used to derive quantitative indentation hardness or reduced-modulus values.

The representative curve selected for neat UHMWPE reached a maximum penetration depth of approximately 37 μm. Representative curves for several TiO_2_-containing formulations exhibited lower maximum penetration depths, including approximately 31.62 μm for 0.1 wt% white TiO_2_, 27.35 μm for 1.0 wt% white TiO_2_, 29.79 μm for 0.1 wt% black TiO_2_, and 34.31 μm for 0.5 wt% black TiO_2_.

In contrast, the representative curves obtained for the 0.8 wt% white- and black-TiO_2_ formulations reached substantially larger maximum penetration depths of approximately 80.65 and 73.58 μm, respectively. Because these values correspond to representative local indentation responses rather than statistically derived formulation-level means, they should not be interpreted as quantitative evidence that the 0.8 wt% formulations possess lower bulk mechanical resistance than the other materials.

The representative load–displacement curves exhibited substantial variation in local penetration behavior among the investigated formulations, without an evident monotonic relationship with TiO_2_ concentration. Because quantitative formulation-level comparisons of penetration depth were not performed, these observations should not be interpreted as statistically supported differences in indentation resistance among formulations. Consequently, the instrumented-indentation measurements are regarded as complementary descriptive evidence of local load–penetration behavior rather than as an independent quantitative measure of nanocomposite hardness or modulus.

### 3.7. Tribological Performance

#### 3.7.1. Volumetric Wear Loss and Specific Wear Rate

The volumetric wear loss and corresponding specific wear rate obtained under dry ball-on-disc conditions are summarized in Table 1 and Table 2. The specific wear rate, *k*, was calculated according to Equation (6).

(6) $$k=\frac{V}{F_{N}L}$$

where *V* is the volumetric wear loss, *F_N_* = 30 is the applied normal load, and *L* = 530 m is the total sliding distance.

Within the white-TiO_2_ series, the lowest measured mean wear volume and specific wear rate were obtained for the 0.8 wt% composition, with values of 2.30 ± 0.04 mm^3^ and (1.45 ± 0.03) × 10^−4^ mm^3^ N^−1^ m^−1^, respectively. The 1.0 wt% white-TiO_2_ composition produced values close to those of neat UHMWPE, whereas the 0.1–0.5 wt% compositions exhibited higher mean wear losses.

The PLIL-modified black-TiO_2_ series exhibited its lowest measured mean wear volume at 0.5 wt%. This composition exhibited a wear volume of 2.06 ± 0.02 mm^3^ and a specific wear rate of (1.30 ± 0.01) × 10^−4^ mm^3^ N^−1^ m^−1^. These mean values were approximately 13% lower than those of neat UHMWPE; the statistical significance of the wear-volume difference is evaluated in Section 3.7.2. In contrast, the 0.1 and 0.3 wt% black-TiO_2_ nanocomposites exhibited higher mean wear losses, whereas the 0.8 and 1.0 wt% compositions produced values close to those of neat UHMWPE.

The observed mean wear values exhibited a non-monotonic pattern across the investigated concentrations for both nanoparticle conditions, with the lowest measured mean wear occurring at different filler concentrations for the untreated white- and PLIL-modified black-TiO_2_ series.

**Figure 10:**
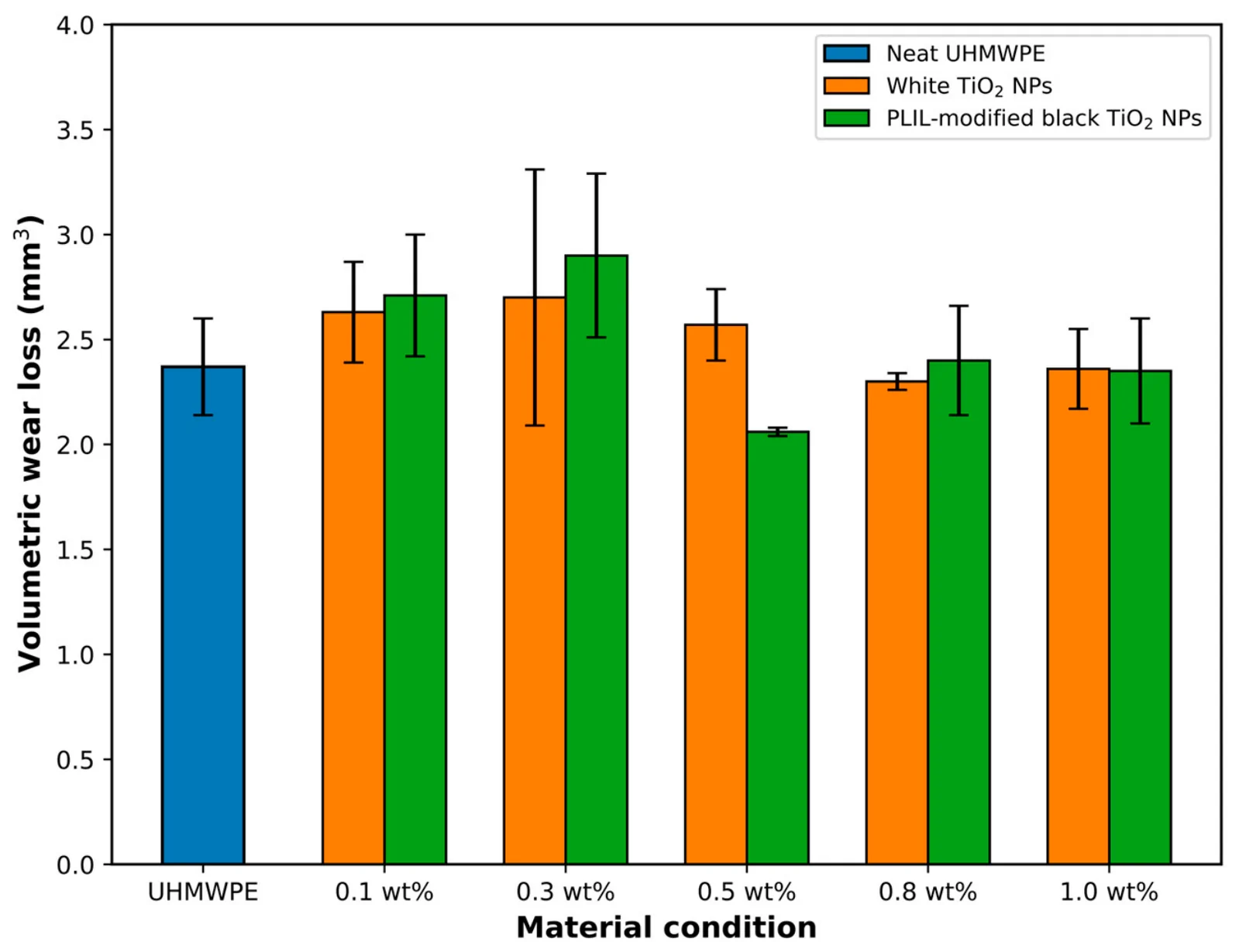
Volumetric wear loss of neat UHMWPE and UHMWPE nanocomposites containing untreated white and PLIL-modified black TiO_2_ under dry ball-on-disc testing. Data are presented as mean ± standard deviation from three independently tested specimens per formulation (*n* = 3).

Figure 10 compares the volumetric wear loss of all material conditions.

#### 3.7.2. Friction Evolution During Sliding

**Figure 11:**
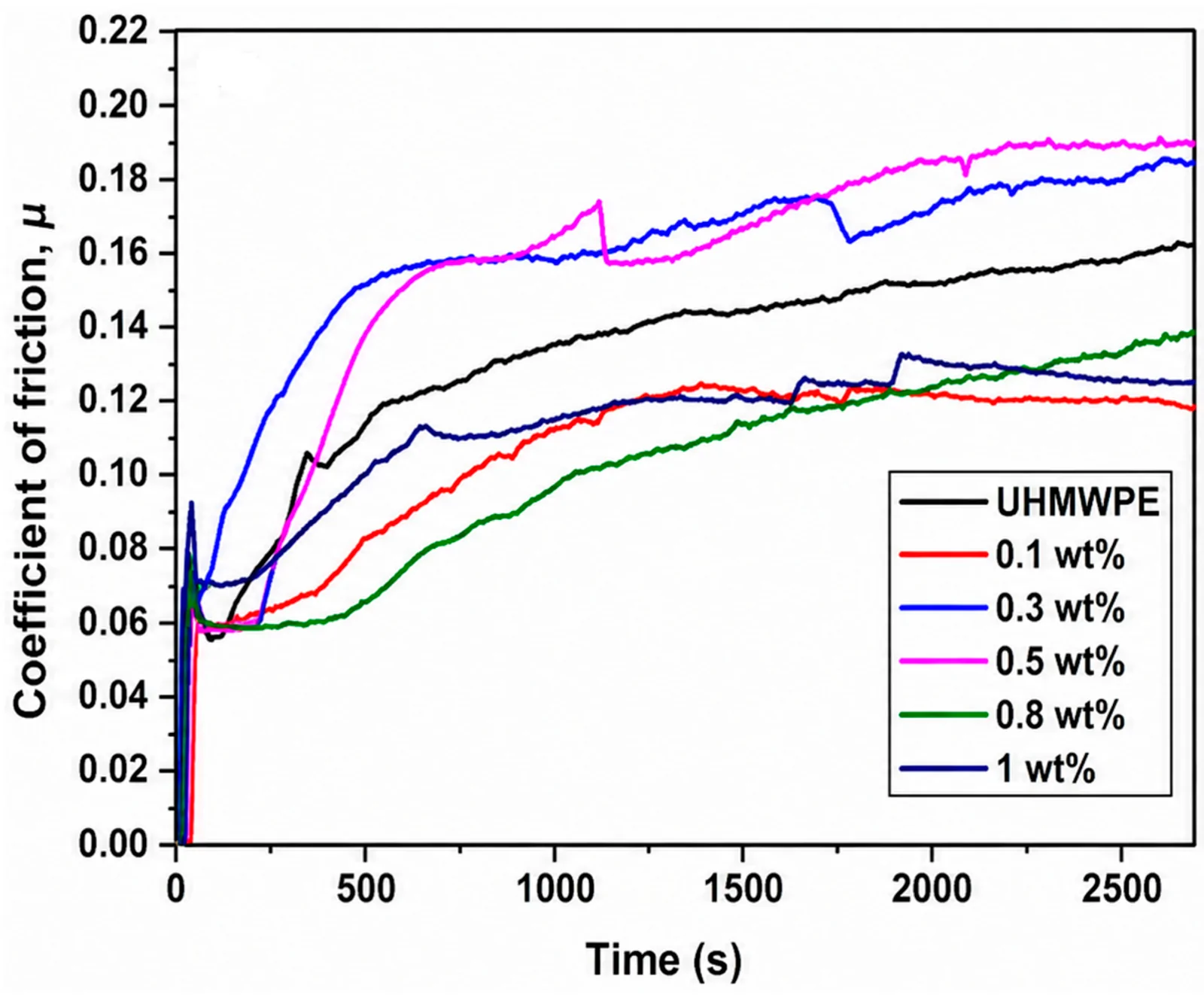
Coefficient of friction as a function of sliding time for neat UHMWPE and nanocomposites containing untreated white TiO_2_.

**Figure 12:**
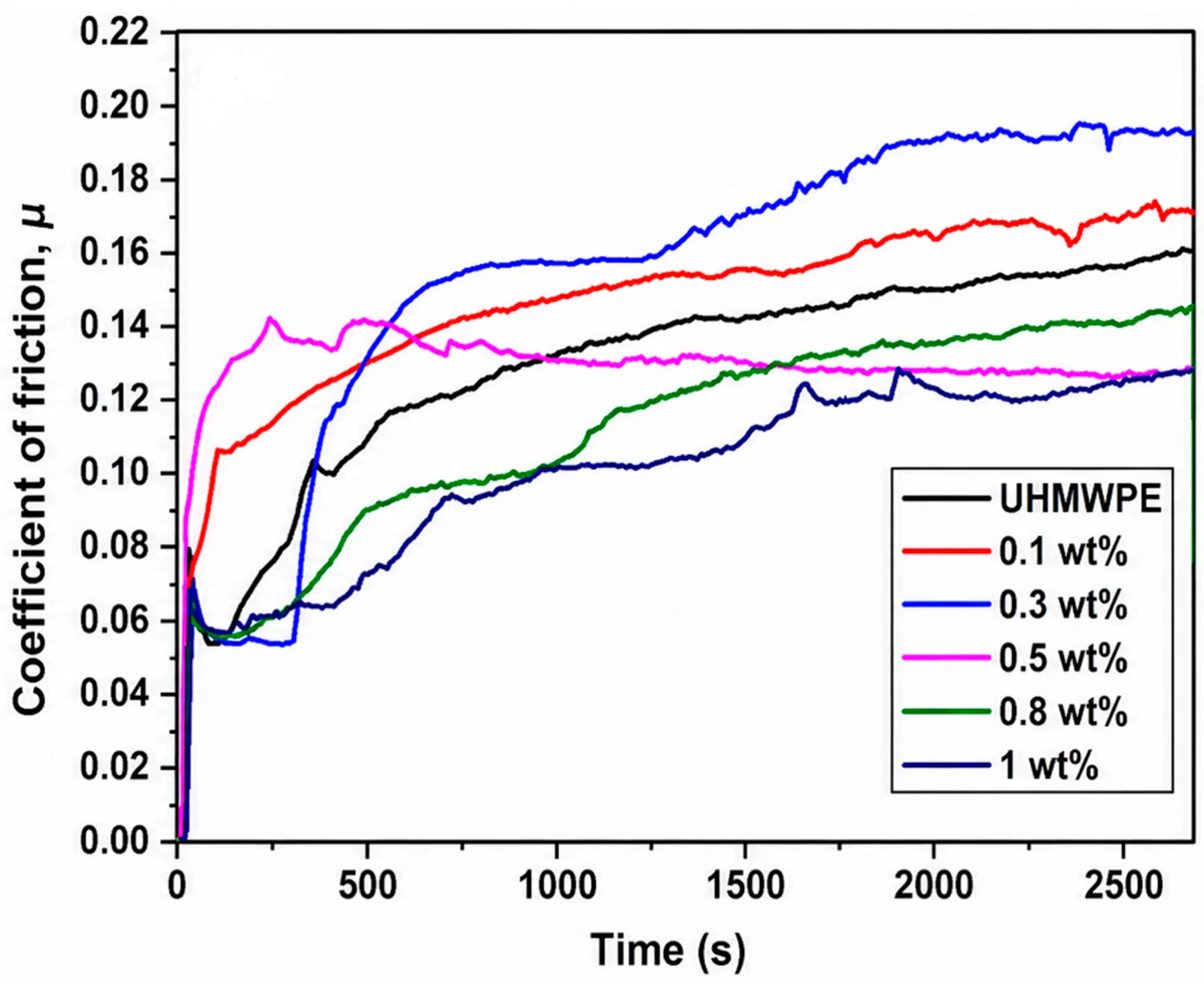
Coefficient of friction as a function of sliding time for neat UHMWPE and nanocomposites containing PLIL-modified black TiO_2_.

Figure 11 and Figure 12 show the temporal evolution of the coefficient of friction for neat UHMWPE and the nanocomposites containing untreated white and PLIL-modified black TiO_2_, respectively. All material conditions exhibited an initial transient or running-in response followed by a composition-dependent evolution of friction during continued sliding. As a common and strictly invariant steady-state regime could not be unambiguously identified for all formulations, quantitative comparison of the friction response was based on the mean terminal coefficient of friction (μ_terminal_), calculated over the same final 400–530 m sliding interval (approximately 2040–2700 s) for each independent test. This terminal interval was used as a standardized comparison window and should not be interpreted as a true steady-state friction regime.

Neat UHMWPE exhibited a mean terminal coefficient of friction of 0.156 ± 0.006. The untreated white-TiO_2_ nanocomposites showed a markedly non-monotonic concentration dependence, with μ_terminal_ values of 0.120 ± 0.005, 0.179 ± 0.007, 0.188 ± 0.008, 0.130 ± 0.005, and 0.127 ± 0.006 for TiO_2_ concentrations of 0.1, 0.3, 0.5, 0.8, and 1.0 wt%, respectively. Thus, within the white-TiO_2_ series, the lowest mean terminal friction coefficient was obtained at 0.1 wt% TiO_2_, whereas the highest value occurred at 0.5 wt% TiO_2_.

The PLIL-modified black-TiO_2_ nanocomposites likewise exhibited a non-monotonic friction response. Mean μ_terminal_ values of 0.169 ± 0.007, 0.192 ± 0.009, 0.128 ± 0.006, 0.141 ± 0.006, and 0.124 ± 0.005 were obtained for 0.1, 0.3, 0.5, 0.8, and 1.0 wt% black TiO_2_, respectively. The highest mean terminal friction coefficient within this series occurred at 0.3 wt%, whereas the lowest value was measured for the 1.0 wt% black-TiO_2_ formulation. The mean terminal coefficients of friction for all material conditions are summarized in Table 3.

Notably, the 0.5 wt% PLIL-modified black-TiO_2_ nanocomposite, which exhibited the lowest measured mean volumetric wear loss among the investigated formulations (2.06 ± 0.02 mm^3^), did not exhibit the lowest mean terminal coefficient of friction. Its μ_terminal_ value was 0.128 ± 0.006, whereas the lowest mean terminal friction coefficients were measured for 0.1 wt% white TiO_2_ (0.120 ± 0.005) and 1.0 wt% black TiO_2_ (0.124 ± 0.005). Thus, the formulation exhibiting the lowest measured mean material loss did not coincide with the formulation exhibiting the lowest terminal friction response. Statistical comparisons of these responses are presented in Section 3.8.3.

#### 3.7.3. Worn-Surface Morphology

**Figure 13:**
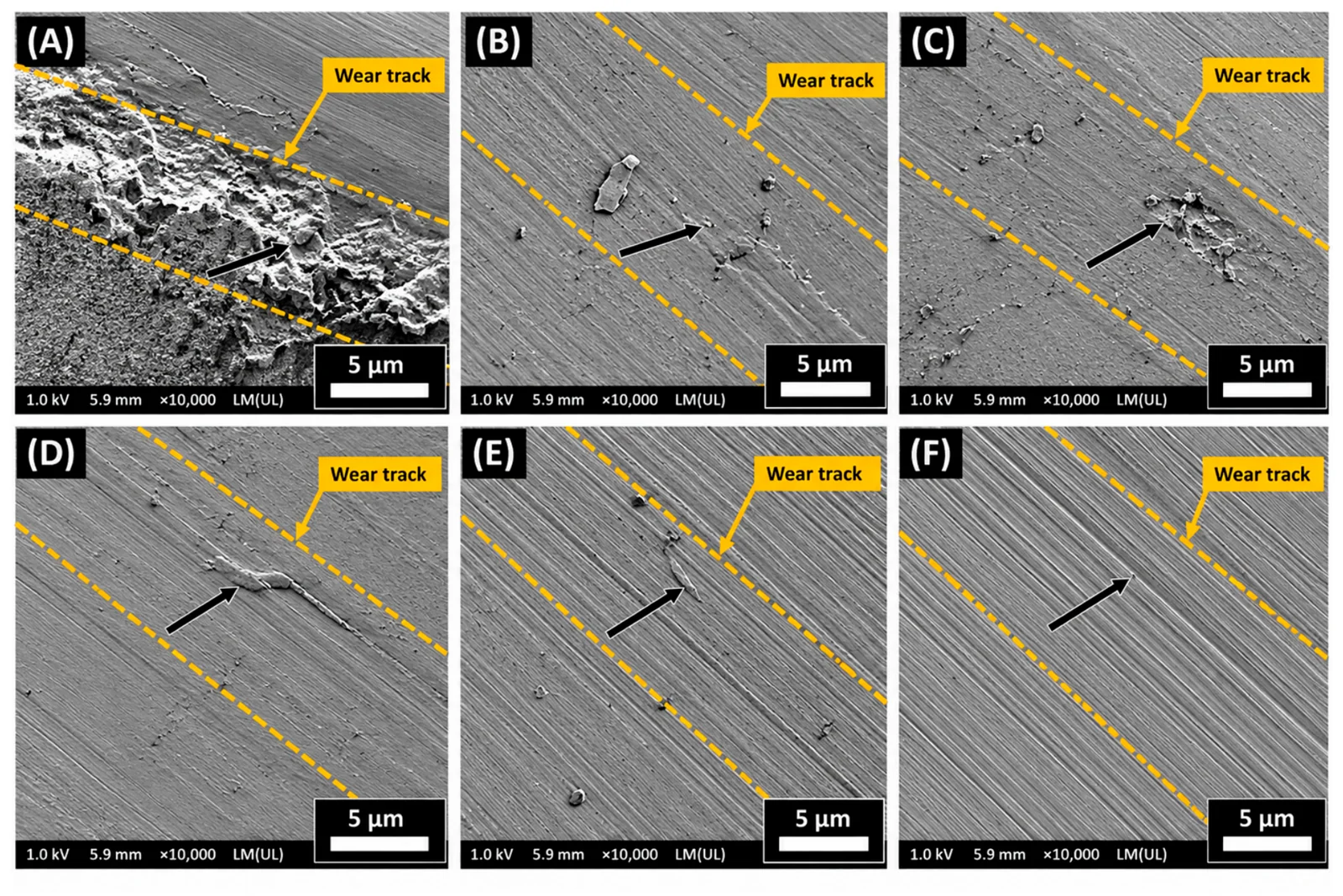
Representative SEM images of the wear tracks of UHMWPE nanocomposites containing untreated white TiO_2_: (**A**) neat UHMWPE, (**B**) 0.1 wt%, (**C**) 0.3 wt%, (**D**) 0.5 wt%, (**E**) 0.8 wt%, and (**F**) 1.0 wt%. The yellow dashed lines delimit the wear-track regions, while the yellow arrows indicate the wear tracks. The black arrows highlight representative surface features associated with the wear process, including localized material deformation, surface grooves, and regions of material accumulation or detachment.

**Figure 14:**
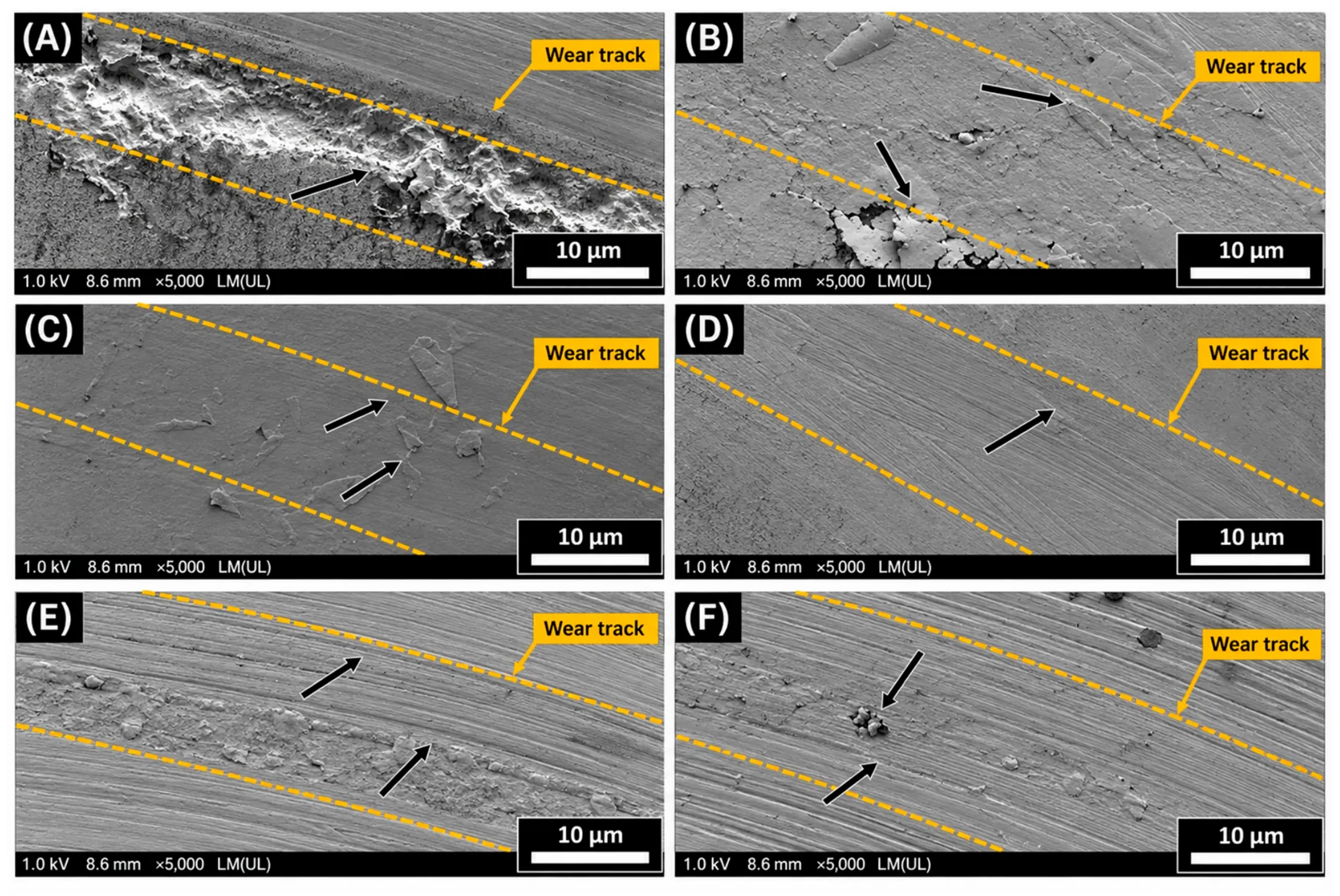
Representative SEM images of the wear tracks of UHMWPE nanocomposites containing PLIL-modified black TiO_2_: (**A**) neat UHMWPE, (**B**) 0.1 wt%, (**C**) 0.3 wt%, (**D**) 0.5 wt%, (**E**) 0.8 wt%, and (**F**) 1.0 wt%. The yellow dashed lines delimit the wear-track regions, while the yellow arrows indicate the wear tracks. The black arrows highlight representative surface features associated with the wear process, including localized material deformation, surface grooves, and regions of material accumulation or detachment.

Figure 13 and Figure 14 show representative SEM micrographs of the worn surfaces after dry ball-on-disc testing. The SEM examination of the wear tracks revealed clear differences in surface morphology between neat UHMWPE and the TiO_2_-containing nanocomposites. Neat UHMWPE exhibited pronounced surface disruption and plastic deformation, characterized by deformed regions and grooves predominantly oriented along the sliding direction. The TiO_2_-containing formulations exhibited composition-dependent variations in the extent and morphology of surface damage, including longitudinal grooves, locally deformed regions, and surface irregularities.

Within the untreated white-TiO_2_ series, the appearance of the worn surfaces varied with nanoparticle concentration. Several formulations exhibited longitudinal grooves and localized surface disruption, while the higher TiO_2_ contents, particularly 0.8 and 1.0 wt%, showed comparatively more pronounced abrasive-like surface features. Nevertheless, the apparent severity of these morphological features did not vary monotonically with TiO_2_ concentration. Accordingly, the SEM observations should be considered as qualitative morphological evidence and interpreted together with the specimen-level wear and friction measurements rather than as an independent measure of tribological performance.

The PLIL-modified black-TiO_2_ nanocomposites likewise exhibited concentration-dependent differences in worn-surface morphology. The observed surfaces ranged from comparatively mild surface modification to more pronounced longitudinal grooving and localized disruption. As in the untreated white-TiO_2_ series, the apparent severity of the worn-surface features did not follow a simple monotonic dependence on TiO_2_ concentration, indicating that increasing nanoparticle content did not produce a correspondingly monotonic change in the visible surface damage.

Of particular interest, the 0.5 wt% PLIL-modified black-TiO_2_ nanocomposite, which exhibited the lowest measured mean volumetric wear loss and specific wear rate among the investigated formulations, displayed comparatively mild visible surface damage relative to the more severely grooved conditions. Its mean wear volume was 2.06 ± 0.02 mm^3^, corresponding to a specific wear rate of approximately 1.30 × 10^−4^ mm^3^ N^−1^ m^−1^, compared with 2.37 ± 0.23 mm^3^ and approximately 1.49 × 10^−4^ mm^3^ N^−1^ m^−1^, respectively, for neat UHMWPE. However, the approximately 13.1% numerical reduction in mean wear volume relative to neat UHMWPE was not statistically significant after Dunnett adjustment (*p* = 0.739). Therefore, the comparatively mild worn-surface morphology of this formulation should be interpreted as being consistent with its lower measured mean material loss within the investigated formulation set, rather than as morphological evidence of a statistically demonstrated improvement in wear resistance relative to neat UHMWPE.

Notably, the worn-surface appearance did not exhibit a direct one-to-one correspondence with the terminal coefficient of friction. The 0.5 wt% PLIL-modified black-TiO_2_ formulation, despite exhibiting the lowest measured mean wear volume and comparatively mild visible surface damage, did not exhibit the lowest mean terminal coefficient of friction. Its μ_terminal_ value of 0.128 ± 0.006 was higher than those measured for the 0.1 wt% untreated white-TiO_2_ formulation (0.120 ± 0.005) and the 1.0 wt% PLIL-modified black-TiO_2_ formulation (0.124 ± 0.006). Thus, the formulation exhibiting the lowest measured mean material loss did not coincide with that exhibiting the lowest terminal friction, further demonstrating that worn-surface morphology, friction, and volumetric wear provide complementary rather than interchangeable descriptions of the tribological response.

The present subsection is intentionally restricted to the phenomenological description of the worn surfaces and their direct comparison with the experimentally measured tribological responses. The SEM observations alone do not establish nanoparticle pull-out, third-body abrasion, transfer-layer formation, preferential particle removal, or differences in particle retention because the chemical composition of the wear tracks and generated debris was not independently determined. Consequently, no specific microscopic wear mechanism is assigned from the SEM morphology alone. Section 4.4 integrates the observed worn-surface morphology with the quantitative wear results while explicitly restricting mechanistic interpretation to conclusions supported by the available experimental evidence.

### 3.8. Statistical Comparison of Mechanical and Tribological Responses

Inferential statistical analyses were performed using the independently fabricated specimen as the experimental unit (*n* = 3 per material condition). For the quantitative response variables subjected to inferential analysis (Vickers microhardness, volumetric wear loss, and mean terminal coefficient of friction), technical measurements obtained from the same specimen were averaged, where applicable, to yield a single specimen-level value and were not treated as independent observations. Instrumented-indentation load–displacement measurements were evaluated descriptively and were not included in the inferential analyses.

For the TiO_2_-containing formulations, two-way analysis of variance (ANOVA) was used to evaluate the main effects of nanoparticle state (untreated white TiO_2_ vs. PLIL-modified black TiO_2_), TiO_2_ concentration (0.1, 0.3, 0.5, 0.8, and 1.0 wt%), and their interaction (nanoparticle state × concentration). Neat UHMWPE was excluded from the factorial ANOVA because nanoparticle state is not defined for the unreinforced control. Comparisons of individual TiO_2_-containing formulations against neat UHMWPE were performed separately using Dunnett-adjusted multiple comparisons, as described in Section 2.8.

The factorial ANOVA results are summarized in Table 4. The three evaluated quantitative response variables exhibited distinct statistical dependencies on nanoparticle state, TiO_2_ concentration, and their interaction. In particular, the terminal friction response exhibited a pronounced concentration dependence together with a strong nanoparticle state × concentration interaction, whereas microhardness and volumetric wear loss exhibited different statistical dependencies.

#### 3.8.1. Statistical Analysis of Vickers Microhardness

For Vickers microhardness, two-way ANOVA revealed statistically significant main effects of nanoparticle state (*F*_1,20_ = 37.92, *p* < 0.0001, partial *η*^2^ = 0.655) and TiO_2_ concentration (*F*_4,20_ = 3.68, *p* = 0.0210, partial *η*^2^ = 0.424). In contrast, the nanoparticle state × concentration interaction was not statistically significant (*F*_4,20_ = 1.15, *p* = 0.3617, partial *η*^2^ = 0.187). These results indicate that both nanoparticle state and TiO_2_ concentration influenced the Vickers microhardness response, whereas the concentration-dependent trends of the untreated white- and PLIL-modified black-TiO_2_ series did not differ sufficiently to produce a statistically significant interaction.

Consistent with the descriptive results presented in Section 3.6.1, the untreated white-TiO_2_ series exhibited higher overall microhardness than the PLIL-modified black-TiO_2_ series across the investigated concentration range. The highest mean microhardness was observed for the 1.0 wt% white-TiO_2_ nanocomposite, which reached 4.19 ± 0.12 HV0.05 compared with 3.34 ± 0.11 HV0.05 for neat UHMWPE, corresponding to an increase of approximately 25%.

Dunnett-adjusted comparisons against neat UHMWPE indicated that all investigated TiO_2_-containing formulations exhibited significantly higher Vickers microhardness than the unreinforced control (*p* < 0.05), although the magnitude of the increase varied with nanoparticle state and concentration. Therefore, the relatively close absolute microhardness values observed among some formulations should not be interpreted as evidence of an absence of filler influence. Rather, the specimen-level statistical analysis demonstrates a measurable effect of TiO_2_ incorporation within the investigated low-filler regime. At the same time, the absence of a statistically significant nanoparticle state × concentration interaction indicates that the two nanoparticle states did not exhibit sufficiently different concentration-dependent trends to establish distinct hardening trajectories. Accordingly, the mechanical response is most appropriately described as a statistically significant but moderate and formulation-dependent increase in Vickers microhardness rather than as a large or monotonic hardening effect with increasing TiO_2_ content.

#### 3.8.2. Statistical Analysis of Wear Performance

For volumetric wear loss, TiO_2_ concentration exhibited a statistically significant main effect (*F*_4,20_ = 3.42, *p* = 0.0277, partial *η*^2^ = 0.406). In contrast, neither nanoparticle state (*F*_1,20_ = 0.068, *p* = 0.7967, partial *η*^2^ = 0.003) nor the nanoparticle state × concentration interaction (*F*_4,20_ = 1.36, *p* = 0.2835, partial *η*^2^ = 0.214) was statistically significant. Thus, within the investigated experimental domain, TiO_2_ concentration showed a detectable overall effect on volumetric wear loss, whereas nanoparticle state, considered as an independent main effect, did not.

Dunnett-adjusted comparisons against neat UHMWPE further showed that none of the individual TiO_2_-containing formulations exhibited a statistically significant difference in volumetric wear loss relative to the unreinforced control (*p* > 0.05 for all comparisons; Table 4). This distinction is particularly important when interpreting the wear response of the 0.5 wt% PLIL-modified black-TiO_2_ nanocomposite. This formulation exhibited the lowest measured mean volumetric wear loss among the investigated material conditions, with a value of 2.06 ± 0.02 mm^3^, compared with 2.37 ± 0.23 mm^3^ for neat UHMWPE. This corresponds to an approximately 13.1% numerical reduction in mean wear volume. Nevertheless, the difference relative to neat UHMWPE was not statistically significant after Dunnett adjustment (*p* = 0.739). Accordingly, this result should be interpreted as the lowest measured mean wear volume within the investigated formulation set rather than as evidence of a statistically significant improvement in wear resistance relative to neat UHMWPE.

The observed mean wear values exhibited a non-monotonic pattern across the investigated concentrations. Within the untreated white-TiO_2_ series, the lowest measured mean wear volume occurred at 0.8 wt% TiO_2_ (2.30 ± 0.04 mm^3^), whereas the PLIL-modified black-TiO_2_ series exhibited its lowest measured mean at 0.5 wt% TiO_2_ (2.06 ± 0.02 mm^3^). Conversely, the 0.1 and 0.3 wt% black-TiO_2_ formulations exhibited higher mean wear volumes of 2.71 ± 0.29 and 2.90 ± 0.39 mm^3^, respectively. Notably, even the largest positive difference relative to neat UHMWPE, observed for the 0.3 wt% black-TiO_2_ formulation (Δ wear volume = +0.53 mm^3^), did not reach statistical significance after Dunnett adjustment (*p* = 0.200). These results therefore do not support a generalized conclusion that PLIL-modified black TiO_2_ universally improves or worsens the wear resistance of UHMWPE. Instead, they demonstrate a formulation-dependent wear response, with the lowest measured mean occurring at 0.5 wt% black TiO_2_ within the investigated composition range.

Because the specific wear rate was calculated directly from volumetric wear loss according to *k* = *V*/(*F_N_L*), and the same normal load and sliding distance were used for all specimens, the specific wear rate represents a constant positive linear transformation of volumetric wear loss. Consequently, a separate inferential analysis of specific wear rate would yield the same *F* statistics and *p*-values as the corresponding analysis of volumetric wear loss. A separate ANOVA for specific wear rate was therefore not performed because it would provide statistically redundant information.

#### 3.8.3. Statistical Analysis of the Friction Response

The mean terminal coefficient of friction (μ_terminal_) exhibited a pronounced concentration-dependent response. Two-way ANOVA revealed a highly significant main effect of TiO_2_ concentration (*F*_4,20_ = 76.23, *p* < 0.0001, partial *η*^2^ = 0.938) and a highly significant nanoparticle state × concentration interaction (*F*_4,20_ = 55.28, *p* < 0.0001, partial *η*^2^ = 0.917). In contrast, the overall main effect of nanoparticle state was not statistically significant (*F*_1,20_ = 0.704, *p* = 0.4113, partial *η*^2^ = 0.034).

The significant interaction indicates that the effect of TiO_2_ concentration on the terminal friction response depended strongly on whether the reinforcement consisted of untreated white or PLIL-modified black TiO_2_. Accordingly, although nanoparticle state alone did not produce a statistically significant overall shift in μ_terminal_ when averaged across all investigated concentrations, the two nanoparticle conditions exhibited markedly different concentration-dependent friction responses. This behavior is consistent with the non-monotonic trends observed in Figure 11 and Figure 12 and summarized in Table 3.

Dunnett-adjusted comparisons against neat UHMWPE (μ_terminal_ = 0.156 ± 0.006) further demonstrated the formulation-dependent nature of the friction response (Table 5). Within the untreated white-TiO_2_ series, the 0.1 wt% formulation exhibited a significantly lower mean terminal coefficient of friction than neat UHMWPE (*p* < 0.001), whereas the 0.3 and 0.5 wt% formulations exhibited significantly higher values (*p* = 0.002 and *p* < 0.001, respectively). At higher white-TiO_2_ contents, the 0.8 and 1.0 wt% formulations again exhibited significantly lower mean terminal coefficients of friction than neat UHMWPE (both *p* < 0.001).

For the PLIL-modified black-TiO_2_ series, the 0.3 wt% formulation exhibited a significantly higher μ_terminal_ than neat UHMWPE (*p* < 0.001), whereas the 0.5 and 1.0 wt% formulations exhibited significantly lower values (both *p* < 0.001). In contrast, the differences observed for the 0.1 and 0.8 wt% black-TiO_2_ formulations did not reach statistical significance after Dunnett adjustment (*p* = 0.137 and *p* = 0.065, respectively).

Taken together, these results demonstrate that the terminal friction response was strongly dependent on TiO_2_ concentration and its interaction with nanoparticle condition, whereas nanoparticle state alone did not produce a significant overall effect across the investigated concentration range. Importantly, neither nanoparticle series exhibited a monotonic relationship between TiO_2_ concentration and μ_terminal_. These findings therefore demonstrate a formulation-specific friction response under the investigated dry-sliding conditions and emphasize that the effect of PLIL modification on terminal friction cannot be interpreted independently of nanoparticle concentration.

#### 3.8.4. Decoupling Among Hardness, Friction, and Wear

Dunnett adjustment (*p* = 0.739). In contrast, its mean terminal coefficient of friction (μ_terminal_ = 0.128 ± 0.006) was significantly lower than that of neat UHMWPE (0.156 ± 0.006; Dunnett-adjusted *p* < 0.001). Nevertheless, this formulation did not exhibit the lowest μ_terminal_ among the investigated materials. The lowest mean terminal coefficient of friction was obtained for the 0.1 wt% white-TiO_2_ formulation (0.120 ± 0.005), followed by the 1.0 wt% black-TiO_2_ formulation (0.124 ± 0.005).

The absence of a one-to-one correspondence between terminal friction and volumetric wear is further illustrated by the 0.1 wt% white-TiO_2_ formulation. Although this formulation exhibited the lowest mean μ_terminal_ (0.120 ± 0.005), significantly below that of neat UHMWPE (Dunnett-adjusted *p* < 0.001), its mean volumetric wear loss (2.63 ± 0.24 mm^3^) was numerically higher than that of the unreinforced polymer (2.37 ± 0.23 mm^3^), although this wear difference was not statistically significant after Dunnett adjustment (*p* = 0.868). Conversely, the 0.5 wt% black-TiO_2_ formulation combined a terminal friction coefficient higher than the minimum observed among the investigated formulations with the lowest measured mean wear volume. Similarly, the 1.0 wt% black-TiO_2_ formulation exhibited a low μ_terminal_ of 0.124 ± 0.005 but a mean wear volume of 2.35 ± 0.25 mm^3^, which was comparable in magnitude to that of neat UHMWPE and was not statistically different from the control. These observations demonstrate that a lower terminal coefficient of friction did not necessarily correspond to a lower volumetric wear loss under the investigated dry-sliding conditions.

A similar decoupling was evident between Vickers microhardness and volumetric wear. The highest mean microhardness was obtained for the 1.0 wt% white-TiO_2_ nanocomposite (4.19 ± 0.12 HV0.05), representing an approximately 25% increase relative to neat UHMWPE, whereas the lowest measured mean volumetric wear loss occurred for the 0.5 wt% PLIL-modified black-TiO_2_ nanocomposite. Thus, the formulation exhibiting the highest Vickers microhardness was not the formulation exhibiting the lowest measured mean wear volume. These results demonstrate that increased microhardness alone was insufficient to predict the relative wear response of the investigated TiO_2_/UHMWPE nanocomposites.

Taken together, these findings demonstrate that Vickers microhardness, terminal friction, and volumetric wear did not exhibit a simple one-to-one correspondence across the investigated formulations. Neither increased microhardness nor reduced terminal friction independently predicted the formulation exhibiting the lowest measured mean wear volume. Rather, the observed tribological response was formulation dependent, with the statistical analysis revealing a strong effect of TiO_2_ concentration and a pronounced nanoparticle state × concentration interaction for terminal friction, together with a significant overall concentration effect on volumetric wear. The response of the 0.5 wt% PLIL-modified black-TiO_2_ formulation therefore represents a concentration-specific combination of mechanical and tribological characteristics rather than a wear improvement attributable solely to maximum hardness or minimum friction. Potential contributions from nanoparticle retention or pull-out, matrix continuity, third-body processes, transfer-layer development, and worn-surface evolution are considered as mechanistic hypotheses in Section 4; none of these mechanisms was directly quantified in the present study.

## 4. Discussion

### 4.1. PLIL-Induced Morphological and Phase Evolution of TiO_2_

The combined FESEM, XRD, and Raman results show that pulsed laser irradiation in liquid (PLIL) produced marked changes in both the morphology and crystalline-phase distribution of the TiO_2_ reinforcement. Untreated white TiO_2_ exhibited predominantly irregular, angular, and faceted particles, whereas the PLIL-modified material contained a visibly larger proportion of rounded and nearly spherical particles. These morphological changes are consistent with prior observations for PLIL-treated TiO_2_ nanoparticles reported in Ref. [29]. Such morphological restructuring has been associated with rapid laser–particle energy transfer, transient localized heating, particle restructuring, partial melting, and subsequent resolidification [29]. Because particle temperature, transient melting, and resolidification kinetics were not directly monitored in the present experiments, these processes should be regarded as literature-supported mechanisms consistent with the observed morphological evolution rather than as directly demonstrated transformation pathways.

The morphological modification was accompanied by a measurable redistribution of the TiO_2_ crystalline phases. Quantitative XRD analysis indicated that untreated white TiO_2_ contained approximately 56% anatase and 44% rutile, whereas the PLIL-modified black TiO_2_ contained approximately 41% anatase and 59% rutile. Thus, PLIL did not produce complete anatase-to-rutile transformation but shifted the crystalline-phase balance from an anatase-dominant to a rutile-dominant assemblage. This change is consistent with previous reports describing laser-induced anatase-to-rutile transformation under sufficiently energetic irradiation conditions [29]. The present results therefore indicate that PLIL promoted a substantial redistribution of the anatase/rutile phase balance while preserving both TiO_2_ polymorphs.

Raman spectroscopy provided complementary spectroscopic evidence for this phase evolution. Both untreated and PLIL-modified TiO_2_ retained vibrational features attributable to anatase and rutile, whereas the PLIL-modified material exhibited more evident rutile-related contributions, particularly near 445 and 612 cm^−1^ [29]. The agreement between the XRD and Raman observations therefore supports the conclusion that PLIL modified the relative anatase/rutile phase distribution in addition to producing the morphological restructuring observed by FESEM.

It is important, however, to distinguish these experimentally observed PLIL-induced modifications from other physicochemical characteristics commonly associated with black TiO_2_. Depending on the synthesis route and treatment conditions, darkened TiO_2_ materials have been associated with oxygen vacancies, Ti^3+^ species, surface disorder, hydrogenation, altered electronic states, and changes in optical absorption. In particular, recent in situ investigations have demonstrated that the hydrogenation behavior of black TiO_2_ can depend on the exposed crystallographic facets, highlighting the complexity of its defect-related physicochemical evolution [36]. However, none of these defect-related characteristics were directly quantified in the present study. XRD and Raman spectroscopy establish the coexistence and redistribution of anatase and rutile crystalline phases, whereas FESEM demonstrates substantial morphological restructuring, but these techniques do not independently determine the chemical or electronic origin of the black coloration.

Accordingly, the term “PLIL-modified black TiO_2_” is used in the present work as an operational descriptor of the visibly darkened TiO_2_ obtained after PLIL treatment rather than as evidence of a specific defect chemistry. The present results do not establish whether the observed darkening originates from Ti^3+^ formation, oxygen vacancies, surface disorder, changes in electronic structure, or a combination of these effects. Determination of the origin of the black coloration would require complementary defect-sensitive characterization, such as X-ray photoelectron spectroscopy of the Ti 2p and O 1s regions, electron paramagnetic resonance, and/or UV–Vis diffuse-reflectance spectroscopy.

Furthermore, because PLIL simultaneously altered particle morphology and the anatase/rutile phase distribution, the present experimental design does not allow the individual contribution of these variables to the mechanical and tribological behavior of the UHMWPE nanocomposites to be isolated. Therefore, the subsequent differences observed between composites containing untreated white and PLIL-modified black TiO_2_ should be attributed to the combined experimentally observed physicochemical modification produced by PLIL, particularly the changes in particle morphology and anatase/rutile phase distribution, rather than to unverified Ti^3+^-, oxygen-vacancy-, or defect-mediated mechanisms. Similarly, these differences cannot be attributed exclusively to particle morphology or crystalline-phase composition because both characteristics changed simultaneously. This distinction is particularly relevant given the non-monotonic and property-dependent responses observed for microhardness, friction, and wear, which indicate that the effect of PLIL modification cannot be interpreted independently of nanoparticle concentration.

### 4.2. Oxidative Stability and Mechanical Response of TiO_2_/UHMWPE Nanocomposites

The FTIR results indicate that the selected hot-pressing conditions did not produce detectable carbonyl-type oxidation of the UHMWPE matrix. No distinct absorption feature was observed in the approximately 1720–1730 cm^−1^ region, commonly associated with carbonyl-containing oxidation products in processed or aged UHMWPE [12,37,38]. Within the detection capability of the FTIR measurements, these results indicate that hot pressing did not induce measurable carbonyl-type oxidative modification of the polymer matrix. Consequently, extensive processing-induced oxidation is unlikely to be the primary factor responsible for the differences subsequently observed in the mechanical and tribological responses. Nevertheless, the absence of a detectable carbonyl absorption band should not be interpreted as evidence for the complete absence of all possible oxidative or chemical modifications below the sensitivity of the analytical method.

The Vickers microhardness results demonstrate that incorporation of relatively small amounts of TiO_2_ can measurably modify the resistance of UHMWPE to localized surface deformation. Although the absolute microhardness values of the investigated formulations remained relatively close, their numerical proximity should be distinguished from statistical equivalence. Specimen-level analysis demonstrated significant main effects of both nanoparticle state (*F*_1,20_ = 37.92, *p* < 0.0001, partial *η*^2^ = 0.655) and TiO_2_ concentration (*F*_4,20_ = 3.68, *p* = 0.0210, partial *η*^2^ = 0.424), whereas the nanoparticle state × concentration interaction was not statistically significant (*F*_4,20_ = 1.15, *p* = 0.3617, partial *η*^2^ = 0.187). Moreover, Dunnett-adjusted comparisons against neat UHMWPE showed that all investigated TiO_2_-containing formulations exhibited significantly higher Vickers microhardness than the unreinforced control (*p* < 0.05).

The magnitude of this effect should nevertheless be interpreted in the context of the deliberately low filler contents investigated. Neat UHMWPE exhibited a mean microhardness of 3.34 ± 0.11 HV0.05, whereas the highest mean value, obtained for the 1.0 wt% untreated white-TiO_2_ nanocomposite, was 4.19 ± 0.12 HV0.05, corresponding to an increase of approximately 25%. The 0.8 wt% untreated white-TiO_2_ formulation similarly reached 4.07 ± 0.15 HV0.05. Thus, the present data support a statistically significant but moderate hardening effect within the investigated 0.1–1.0 wt% low-filler regime rather than a large or monotonic increase in surface hardness with increasing TiO_2_ content. The absence of a statistically significant nanoparticle state × concentration interaction further indicates that, although nanoparticle state and concentration independently influenced Vickers microhardness, the concentration-dependent trends of the untreated white- and PLIL-modified black-TiO_2_ series were not sufficiently different to establish statistically distinct hardening trajectories.

From a materials perspective, such a response is consistent with the low nanoparticle concentrations investigated. At 0.1–1.0 wt%, TiO_2_ remains a dispersed reinforcing phase within a mechanically dominant UHMWPE matrix rather than forming a continuous load-bearing ceramic phase. The observed increase in Vickers microhardness may therefore be consistent with local restriction of polymer deformation and stress transfer in the vicinity of sufficiently dispersed and mechanically retained nanoparticles. Conversely, local variations in nanoparticle distribution, particle-rich and matrix-rich regions, agglomeration, or differences in particle–matrix interaction could limit the magnitude and uniformity of the reinforcement effect. These considerations are consistent with previous investigations highlighting the critical influence of nanofiller dispersion and agglomeration on the mechanical performance of polymer nanocomposites [39]. However, because quantitative nanoparticle-dispersion mapping, particle–matrix interfacial characterization, and spatially resolved structure–property correlations were not performed in the present study, these mechanisms should be regarded as physically plausible interpretations rather than directly demonstrated causes of the measured microhardness response.

The differences between the untreated white- and PLIL-modified black-TiO_2_ series should likewise be interpreted cautiously. PLIL treatment produced experimentally demonstrated changes in nanoparticle morphology and anatase/rutile phase balance, but the present characterization does not permit the individual contributions of morphology, crystalline-phase redistribution, particle or agglomerate dimensions, dispersion, or particle–matrix interfacial behavior to the mechanical response to be separated quantitatively. Consequently, the statistically significant main effect of nanoparticle state establishes that the experimentally defined untreated and PLIL-modified TiO_2_ conditions produced different overall Vickers microhardness responses, but it does not identify a unique physicochemical mechanism responsible for that difference. In particular, the results do not support a generalized conclusion that PLIL modification increases the surface hardness of UHMWPE; instead, the untreated white-TiO_2_ formulations generally exhibited the higher Vickers microhardness values within the investigated composition range.

The instrumented-indentation load–displacement curves provide complementary information regarding local deformation behavior. Several representative TiO_2_-containing curves exhibited lower penetration depths than the representative neat-UHMWPE response, whereas the representative curves for the 0.8 wt% white- and black-TiO_2_ formulations showed substantially greater penetration depths. These observations reveal considerable variability in the local load–penetration response and demonstrate that the instrumented-indentation behavior does not follow a simple monotonic relationship with TiO_2_ concentration or with the specimen-level Vickers microhardness response.

Potential factors contributing to this local variability include spatial differences in nanoparticle distribution, matrix-rich or particle-rich regions, surface topography, consolidation heterogeneity, or other localized microstructural features. However, quantitative cross-sectional compositional mapping, porosity measurements, surface-roughness characterization, and spatially resolved microstructural analysis were not performed. Therefore, none of these factors can presently be identified as the cause of the unusually large penetration depths observed in the representative 0.8 wt% curves.

The instrumented-indentation results should therefore be regarded as complementary descriptive evidence of local load–penetration behavior rather than as an independent quantitative measurement of indentation hardness or elastic modulus. A statistically supported evaluation of indentation-derived mechanical properties would require specimen-level analysis of multiple indentation locations, validated tip-area-function and instrument-compliance calibration, appropriate thermal-drift assessment, contact-area analysis, and consideration of the time-dependent viscoelastic response of UHMWPE. Such analyses were beyond the scope of the present study. Accordingly, direct quantitative comparison between the representative instrumented-indentation curves and the specimen-level Vickers microhardness results should be made cautiously.

Taken together, the mechanical results demonstrate a statistically significant but moderate and formulation-dependent increase in Vickers microhardness following TiO_2_ incorporation, while the representative instrumented-indentation curves reveal substantial variability in local load–penetration behavior. Accordingly, the quantitative mechanical conclusions of the present study are based primarily on the specimen-level Vickers microhardness measurements. The instrumented-indentation results are interpreted as complementary descriptive observations and do not permit formulation-level quantitative conclusions concerning indentation hardness or reduced modulus. Importantly, the observed increase in Vickers microhardness should not by itself be interpreted as evidence of improved tribological performance, because the formulation exhibiting the highest microhardness did not coincide with that exhibiting the lowest measured mean wear volume. This decoupling indicates that the tribological response of TiO_2_/UHMWPE nanocomposites is governed by a more complex interplay among nanoparticle condition, concentration, local mechanical response, interfacial phenomena, and sliding-contact processes, as discussed in the following sections.

### 4.3. Concentration-Dependent Wear and Friction Response

A central finding of the present study is that TiO_2_ incorporation did not produce a monotonic improvement in the wear response of UHMWPE. For the untreated white-TiO_2_ series, the lowest measured mean wear volume occurred at 0.8 wt% (2.30 ± 0.04 mm^3^), whereas the PLIL-modified black-TiO_2_ series exhibited its lowest measured mean at 0.5 wt% (2.06 ± 0.02 mm^3^). The latter corresponded to a specific wear rate of approximately 1.30 × 10^−4^ mm^3^ N^−1^ m^−1^, compared with 2.37 ± 0.23 mm^3^ and approximately 1.49 × 10^−4^ mm^3^ N^−1^ m^−1^ for neat UHMWPE. Thus, the 0.5 wt% black-TiO_2_ formulation exhibited an approximately 13.1% numerical reduction in mean wear volume relative to neat UHMWPE. However, this difference was not statistically significant after Dunnett adjustment (*p* = 0.739). Accordingly, this composition should be regarded as exhibiting the lowest measured mean wear volume within the investigated formulation set, rather than a statistically demonstrated improvement in wear resistance relative to neat UHMWPE.

The statistical results further reinforce the concentration-dependent nature of the wear response. TiO_2_ concentration produced a significant overall effect on volumetric wear loss (*F*_4,20_ = 3.42, *p* = 0.0277, partial *η*^2^ = 0.406), whereas neither nanoparticle state nor the nanoparticle state × concentration interaction was statistically significant. Moreover, none of the individual TiO_2_-containing formulations differed significantly from neat UHMWPE in Dunnett-adjusted comparisons of wear volume. These findings indicate that concentration influenced the overall pattern of wear across the investigated composites, but they do not support a generalized conclusion that either untreated white or PLIL-modified black TiO_2_ consistently improved wear resistance relative to the unreinforced polymer.

The observed non-monotonic concentration dependence is consistent with the complex behavior reported for particle-reinforced UHMWPE, in which tribological performance may depend on the combined effects of reinforcement content, particle distribution, matrix continuity, particle–matrix interactions, particle retention, and the evolution of debris and third bodies during sliding [11,40,41,42,43]. Increasing the concentration of a hard particulate reinforcement therefore does not necessarily result in a continuous reduction in polymer wear. At unfavorable filler contents, local particle-rich regions, agglomeration, or particle detachment may instead contribute to surface damage and material removal [42]. Such mechanisms provide a plausible framework for interpreting the present non-monotonic response; however, their individual contributions cannot be established solely from the wear and friction measurements.

The newly incorporated EDS elemental maps provide direct spatial evidence of Ti-containing features in both the central and edge regions of the 1.0 wt% composite. Importantly, this analysis was performed at the highest TiO_2_ concentration investigated, representing the formulation with the greatest expected susceptibility to localized particle accumulation within the present composition range. No gross Ti-rich segregation was evident within the analyzed fields; instead, Ti-containing signals were spatially distributed within the carbon-rich UHMWPE matrix in both examined regions. This observation indicates that gross micron-scale segregation within the mapped regions is unlikely, by itself, to account for the non-monotonic wear response. Nevertheless, owing to the finite spatial resolution and localized sampling volume inherent to EDS mapping, these results do not exclude sub-resolution nanoparticle agglomeration, local particle-rich domains outside the analyzed fields, or heterogeneity at smaller length scales. Accordingly, particle agglomeration is not invoked here as an experimentally demonstrated origin of the observed wear behavior, and the mechanistic interpretation is restricted to phenomena consistent with the combined EDS, wear, friction, and worn-surface evidence.

At higher nanoparticle concentrations, reduced interparticle spacing may still influence local matrix continuity, particle–matrix interactions, and particle retention during sliding. However, the EDS observations at 1.0 wt% TiO_2_ indicate that the non-monotonic tribological response cannot be attributed simply to gross filler segregation within the analyzed regions. Moreover, because the present EDS analysis characterizes filler distribution in the composite rather than the chemical composition and evolution of particles within the sliding interface, mechanisms involving nanoparticle pull-out, preferential particle removal, third-body abrasion, or transfer-layer development remain unverified. These processes are therefore considered only as possible contributors to the observed tribological behavior rather than experimentally established mechanisms.

The friction results provide an additional perspective on this concentration-dependent behavior. Quantitative comparison was based on the mean terminal coefficient of friction (μ_terminal_) calculated over the common final sliding interval of 400–530 m, rather than on an assumed steady-state coefficient. The lowest mean μ_terminal_ among all investigated formulations was obtained for 0.1 wt% white TiO_2_ (0.120 ± 0.005), followed by 1.0 wt% black TiO_2_ (0.124 ± 0.005). In contrast, the 0.5 wt% black-TiO_2_ formulation, which exhibited the lowest measured mean wear volume, showed a somewhat higher μ_terminal_ of 0.128 ± 0.006. Nevertheless, its terminal friction coefficient remained significantly lower than that of neat UHMWPE (0.156 ± 0.006; Dunnett-adjusted *p* < 0.001). Thus, the formulation exhibiting the lowest measured mean wear volume did not coincide with the formulation exhibiting the lowest terminal friction response.

The statistical behavior of friction further illustrates the importance of nanoparticle concentration. TiO_2_ concentration exerted a highly significant effect on μ_terminal_ (*F*_4,20_ = 76.23, *p* < 0.0001, partial *η*^2^ = 0.938), together with a strong nanoparticle state × concentration interaction (*F*_4,20_ = 55.28, *p* < 0.0001, partial *η*^2^ = 0.917). In contrast, nanoparticle state alone did not produce a significant overall effect when averaged across concentrations (*p* = 0.4113). This interaction indicates that PLIL modification cannot be interpreted as producing a uniform increase or decrease in friction; rather, its effect depended strongly on TiO_2_ concentration. The non-monotonic friction trends of both nanoparticle series are therefore consistent with a formulation-specific evolution of the sliding interface.

The decoupling between friction and wear is particularly evident when individual formulations are compared. The 0.1 wt% white-TiO_2_ nanocomposite exhibited the lowest mean terminal friction coefficient (0.120 ± 0.005), significantly below that of neat UHMWPE (*p* < 0.001), yet its mean wear volume (2.63 ± 0.24 mm^3^) was numerically higher than that of the unreinforced polymer (2.37 ± 0.23 mm^3^) and was not statistically different from the control (*p* = 0.868). Conversely, the 0.5 wt% black-TiO_2_ formulation exhibited a slightly higher terminal friction coefficient but the lowest measured mean wear volume. This comparison provides direct evidence that, under the present dry-sliding conditions, lower terminal friction did not necessarily correspond to lower material loss.

This decoupling is physically plausible because friction and wear, although interrelated, represent distinct tribological responses. Mechanistically, the frictional response under the present dry ball-on-disc conditions is interpreted as a mixed interfacial process governed by polymer–counterbody adhesion, viscoplastic deformation of the UHMWPE surface, and a composition-dependent contribution from ploughing. For neat UHMWPE, interfacial shear against the H13 steel counterbody and deformation of the relatively compliant polymer are considered the principal contributions to friction, consistent with the pronounced plastic deformation and sliding-direction surface features observed after testing. Incorporation of TiO_2_ modifies this balance by altering the near-surface mechanical response, local load support, and resistance to deformation, whereas the more pronounced longitudinal grooves observed for several higher-filler formulations are consistent with an increased ploughing contribution. The evolution of the friction curves with sliding distance further indicates that the contact interface develops dynamically during testing rather than exhibiting an identical invariant friction regime for all formulations. Importantly, although particle entrainment, third-body interactions, and transfer-layer development may additionally influence interfacial shear, these processes were not directly verified through compositional analysis of the wear debris or counterbody surface and are therefore regarded as plausible secondary contributions rather than experimentally established friction mechanisms. Wear volume, in contrast, quantifies irreversible material loss and additionally depends on near-surface mechanical integrity, particle retention, debris generation, and the evolution of the sliding interface [17,18,43]. Consequently, minimizing friction does not necessarily minimize wear. In the present TiO_2_/UHMWPE system, the combined mechanical, friction, wear, and worn-surface results therefore indicate that tribological performance emerges from a concentration-dependent balance of multiple interfacial and near-surface processes rather than from a single controlling property.

Importantly, these concentration-dependent tribological differences should not be attributed to Ti^3+^ species, oxygen vacancies, or other defect-related characteristics potentially associated with black TiO_2_, because such characteristics were not directly established in the present study. Any distinction between the untreated white- and PLIL-modified black-TiO_2_ series should therefore be interpreted within the experimentally demonstrated PLIL-induced changes in the overall physicochemical state of the reinforcement and their interaction with TiO_2_ concentration.

### 4.4. Evidence-Based Interpretation of Worn-Surface Morphology

The SEM observations revealed clear formulation-dependent differences in the morphology of the worn UHMWPE surfaces. Neat UHMWPE exhibited pronounced surface disruption and plastic deformation, including deformed regions and grooves predominantly oriented along the sliding direction. The TiO_2_-containing nanocomposites exhibited varying degrees of longitudinal grooving and surface disruption, with comparatively pronounced abrasive-like features observed for several of the higher-filler formulations, particularly at 0.8 and 1.0 wt%. These observations demonstrate that the morphology and apparent severity of surface damage varied with formulation; however, SEM morphology alone does not establish the specific physical origin of these features or identify a unique dominant wear mechanism.

The morphological observations are most appropriately interpreted in conjunction with the quantitative wear measurements. The 0.5 wt% PLIL-modified black-TiO_2_ nanocomposite exhibited comparatively mild visible surface damage and the lowest measured mean volumetric wear loss among the investigated formulations (2.06 ± 0.02 mm^3^), corresponding to a specific wear rate of approximately 1.30 × 10^−4^ mm^3^ N^−1^ m^−1^. In comparison, neat UHMWPE exhibited a mean wear volume of 2.37 ± 0.23 mm^3^. Although this represents an approximately 13.1% numerical reduction in mean wear volume, the difference was not statistically significant after Dunnett adjustment (*p* = 0.739). Consequently, the comparatively mild worn-surface morphology of the 0.5 wt% PLIL-modified black-TiO_2_ formulation is consistent with its lower measured mean material loss within the investigated formulation set, but it should not be interpreted as demonstrating a statistically significant improvement in wear resistance relative to neat UHMWPE.

Conversely, several formulations exhibiting more pronounced longitudinal grooves and surface disruption did not necessarily show statistically greater volumetric wear than the unreinforced control. This observation further indicates that qualitative SEM appearance and volumetric material loss represent complementary, but not interchangeable, measures of tribological response. The worn-surface micrographs provide spatial information regarding the morphology of surface damage, whereas the wear-volume measurements provide a specimen-level estimate of material loss. Accordingly, mechanistic conclusions should not be inferred from either measurement in isolation.

The longitudinal grooves observed on several worn surfaces are morphologically consistent with ploughing or abrasive-like surface deformation during sliding. However, the present experiments do not establish whether these features resulted from direct interaction with the steel counterbody, detached UHMWPE debris, TiO_2_-containing debris, nanoparticle or agglomerate detachment, or a combination of these processes. Similarly, nanoparticle pull-out, third-body abrasion, transfer-layer formation, and differences in particle retention were not directly demonstrated because the chemical composition of the wear tracks and generated debris was not independently determined. These processes are therefore not assigned as experimentally established wear mechanisms in the present study.

Accordingly, the ploughing contribution considered in the frictional response should be distinguished from abrasive material removal. Ploughing can contribute to tangential resistance through deformation and displacement of the polymer within the sliding contact without necessarily resulting in a proportional increase in net material loss. Therefore, the presence of longitudinal grooves consistent with ploughing should not, by itself, be interpreted as evidence of an equivalent increase in abrasive wear. This distinction is consistent with the experimentally observed absence of a one-to-one relationship among worn-surface morphology, terminal friction coefficient, and volumetric wear loss.

The SEM results support a formulation-dependent evolution of worn-surface morphology but do not provide sufficient evidence to establish a unique causal relationship between TiO_2_ nanoparticle state, filler concentration, and a specific microscopic wear mechanism. Direct confirmation of such relationships would require paired pre- and post-tribological compositional characterization specifically targeted at the polymer test surface, wear track, generated debris, and H13 steel counterbody surface, for example by SEM–EDS and, where chemically informative, complementary spectroscopic analysis. Such measurements would permit direct assessment of nanoparticle retention or removal, material transfer across the sliding interface, and the chemical nature of any tribologically generated surface layer. The present worn-surface observations are therefore interpreted conservatively as morphological evidence that complements, rather than mechanistically explains, the statistically evaluated friction and wear responses.

### 4.5. Processing–Structure–Tribological Property Relationship

**Figure 15:**
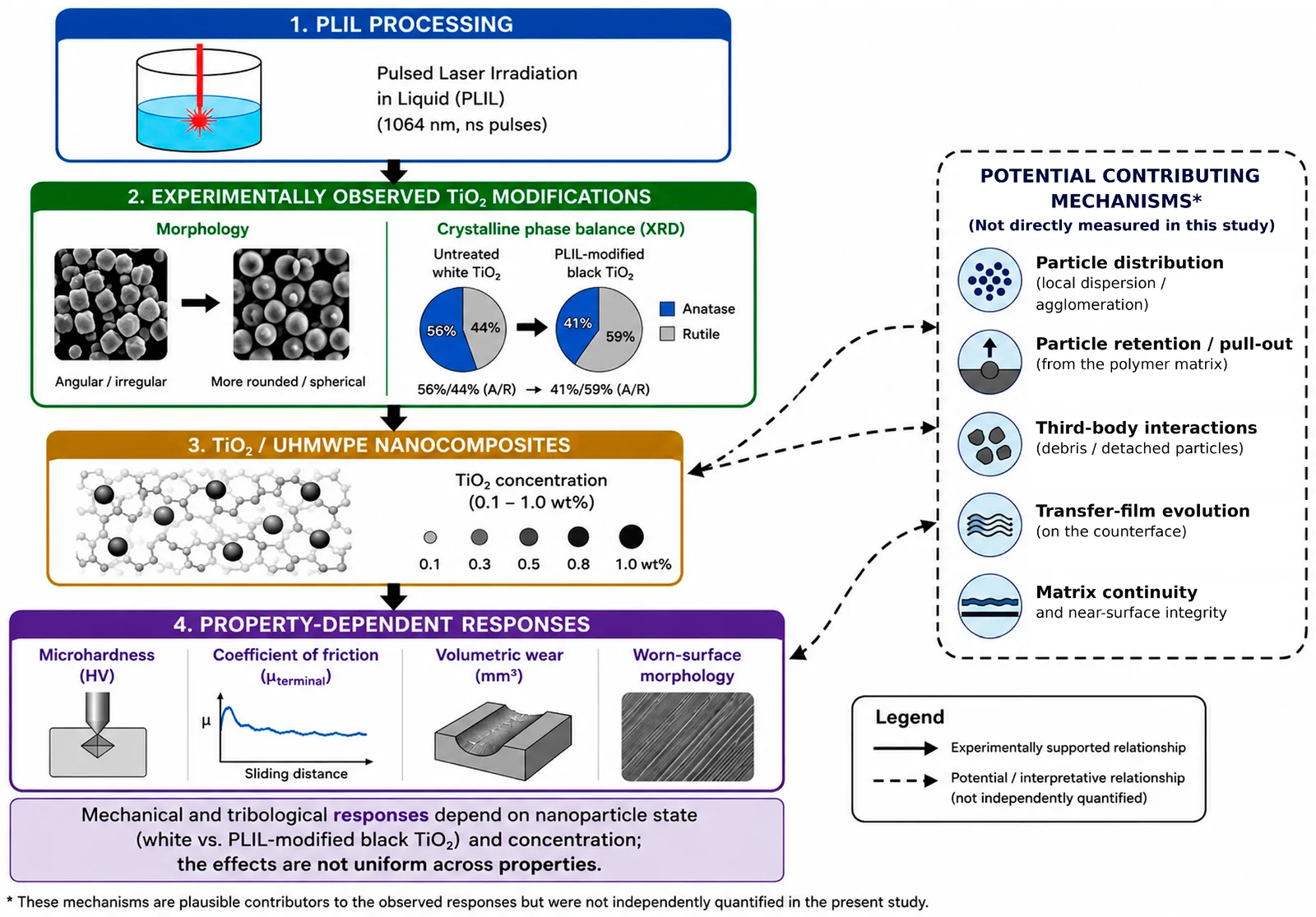
Schematic representation of the processing–structure–response framework linking pulsed laser irradiation in liquid (PLIL)-induced modification of TiO_2_ to the mechanical and tribological behavior of TiO_2_/UHMWPE nanocomposites. PLIL produced experimentally observed changes in TiO_2_ particle morphology and anatase/rutile phase balance, while the subsequent nanocomposite responses depended on both nanoparticle state (untreated white vs. PLIL-modified black TiO_2_) and TiO_2_ concentration (0.1–1.0 wt%). The evaluated responses include Vickers microhardness, mean terminal coefficient of friction (μ_terminal_), volumetric wear loss, and worn-surface morphology. Solid arrows indicate experimentally supported relationships, whereas dashed arrows indicate potential mechanistic contributions (including particle distribution, particle retention/pull-out, third-body interactions, transfer-film evolution, and matrix continuity) that were not independently quantified in the present study. The designation “PLIL-modified black TiO_2_” denotes the visibly darkened PLIL-treated material and does not imply experimentally established Ti^3+^, oxygen vacancies, or a specific defect chemistry.

The combined results establish an experimental sequence connecting PLIL processing with changes in experimentally characterized physicochemical features of the TiO_2_ reinforcement and the subsequent mechanical and tribological responses of the UHMWPE nanocomposites, as schematically summarized in Figure 15. Dashed arrows in Figure 15 denote plausible mechanistic hypotheses which were not directly quantified in the present experiments. This framework should be interpreted as a processing–structure–response relationship supported by the experimentally observed changes, rather than as a complete causal description of all mechanisms governing the tribological behavior.

The experimental results show that PLIL treatment produced a visible darkening of the TiO_2_ powder together with independently characterized changes in particle morphology and crystalline-phase balance. Under the irradiation conditions employed in this study, FESEM revealed a transition from predominantly irregular and angular particles toward a greater prevalence of rounded and nearly spherical particles, whereas quantitative XRD analysis indicated a shift from approximately 56% anatase/44% rutile in untreated white TiO_2_ to approximately 41% anatase/59% rutile after PLIL modification. Raman spectroscopy provided complementary evidence for this phase redistribution. The visible darkening itself should be distinguished from these experimentally established structural and morphological modifications because its chemical and electronic origin was not independently determined. Incorporation of untreated white and PLIL-modified black TiO_2_ into the same UHMWPE matrix subsequently resulted in distinct concentration-dependent mechanical and tribological responses.

The statistical results reinforce the importance of considering nanoparticle state and concentration together. Microhardness was significantly affected by both nanoparticle state and TiO_2_ concentration, although their interaction was not statistically significant. Volumetric wear loss exhibited a significant overall concentration effect, whereas neither nanoparticle state nor the nanoparticle state × concentration interaction was significant. In contrast, the mean terminal coefficient of friction (μ_terminal_) exhibited a pronounced concentration dependence together with a strong nanoparticle state × concentration interaction. These different statistical dependencies demonstrate that PLIL modification did not produce a uniform improvement or deterioration across all measured properties. Rather, the response depended on both the property considered and the TiO_2_ concentration.

Importantly, the present experimental design does not permit the individual contributions of particle morphology and crystalline-phase redistribution to be separated because both characteristics were modified simultaneously by PLIL. Therefore, the lower measured mean wear observed for the 0.5 wt% PLIL-modified black-TiO_2_ formulation should not be attributed exclusively to either its higher rutile fraction or its more rounded particle morphology. Moreover, although this formulation exhibited the lowest measured mean wear volume within the investigated formulation set, its approximately 13.1% numerical reduction relative to neat UHMWPE was not statistically significant after Dunnett adjustment (*p* = 0.739). PLIL should therefore be regarded as a processing route that modifies the overall physicochemical state of the TiO_2_ reinforcement, while the resulting composite response remains strongly dependent on nanoparticle concentration.

Likewise, because Ti^3+^ species, oxygen vacancies, surface disorder, and electronic defect states were not directly characterized, these features cannot be included as experimentally established links between PLIL treatment and the subsequent mechanical or tribological response. Within the present processing–structure–response framework, the experimentally supported PLIL-induced changes are therefore restricted primarily to visible darkening, morphological restructuring, and redistribution of the anatase/rutile crystalline-phase balance.

The absence of a one-to-one correspondence among microhardness, terminal friction, and volumetric wear further demonstrates that the tribological behavior of these nanocomposites cannot be rationalized using a single material parameter. The highest mean microhardness was obtained for 1.0 wt% white TiO_2_ (4.19 ± 0.12 HV), whereas the lowest mean terminal coefficient of friction was obtained for 0.1 wt% white TiO_2_ (μ_terminal_ = 0.120 ± 0.005). In contrast, the lowest measured mean volumetric wear loss occurred for 0.5 wt% PLIL-modified black TiO_2_ (2.06 ± 0.02 mm^3^). Thus, the formulation exhibiting the highest microhardness was not the formulation exhibiting the lowest measured mean wear, and the formulation exhibiting the lowest terminal friction likewise did not exhibit the lowest measured mean wear.

These observations indicate that the tribological response likely emerges from the combined influence of near-surface mechanical behavior, nanoparticle characteristics, polymer-matrix continuity, local particle distribution, and the evolving condition of the sliding interface. Additional processes involving particle retention or pull-out, debris generation, third-body interactions, and transfer-film development may also contribute to the observed response. However, because these mechanisms were not independently quantified in the present study, they should be regarded as plausible mechanistic contributions rather than experimentally demonstrated links within the processing–structure–tribological response framework.

Taken together, the results establish a coherent framework linking PLIL processing to the experimentally observed morphological and crystalline-phase modifications of TiO_2_ and, subsequently, to the concentration-dependent response of the resulting UHMWPE nanocomposites. The principal contribution of this relationship is the demonstration that PLIL-induced modification of the TiO_2_ reinforcement, together with nanoparticle concentration, produces distinct and property-dependent responses in UHMWPE rather than a uniform enhancement in mechanical, frictional, and wear performance [44].

### 4.6. Study Limitations and Implications for Biomedical Bearing Applications

The present results should be interpreted within the experimental scope of the study. Although SEM–EDS mapping provided direct spatial evidence of Ti-containing features in the central and edge regions of the 1.0 wt% composite, nanoscale nanoparticle dispersion and matrix–particle interfacial behavior were not quantitatively resolved, and the chemical composition of the wear tracks, debris, and counterbody surfaces was not independently determined. Consequently, mechanisms involving local particle distribution, particle retention or pull-out, third-body interactions, or transfer-film formation cannot be established from the present data. Similarly, because defect-sensitive characterization was not performed, the chemical or electronic origin of the PLIL-induced black coloration remains unresolved; the term “PLIL-modified black TiO_2_” therefore remains an operational descriptor rather than evidence of a specific defect chemistry.

Wear volume was estimated using a standardized width-based geometrical approach rather than direct three-dimensional profilometry, and the instrumented-indentation curves were used descriptively rather than to derive statistically supported indentation hardness or reduced modulus. In addition, the statistical analysis was based on three independently fabricated specimens per formulation (*n* = 3). These limitations do not invalidate the comparative trends observed under the standardized experimental conditions but constrain the precision and mechanistic interpretation of the measured differences.

Finally, the dry ball-on-disc configuration constitutes a controlled comparative materials-screening test and does not reproduce the multidirectional loading, lubrication, counterface conditions, or biological environment of an artificial joint. Accordingly, the present results should not be extrapolated directly to in vivo performance. Previous studies on functionalized UHMWPE-based nanocomposites have demonstrated that biological compatibility must be evaluated independently of improvements in material properties, emphasizing the importance of dedicated biological characterization when considering biomedical applications [45]. Therefore, additional investigations addressing cytocompatibility, particle release, biological responses to wear debris, and tribological behavior under physiologically relevant conditions would be required before the present TiO_2_/UHMWPE formulations could be considered for orthopedic bearing applications. Within this scope, the study demonstrates that PLIL-induced modification of TiO_2_ morphology and anatase/rutile phase balance, together with nanoparticle concentration, influences the mechanical and tribological response of UHMWPE. The 0.5 wt% PLIL-modified black-TiO_2_ formulation exhibited the lowest measured mean wear volume within the investigated formulation set, although its reduction relative to neat UHMWPE was not statistically significant.

## 5. Conclusions

PLIL produced experimentally demonstrated changes in TiO_2_ morphology and crystalline-phase balance, increasing the prevalence of rounded particles and shifting the phase composition from approximately 56% anatase/44% rutile to 41% anatase/59% rutile. The resulting darkened powder is therefore designated operationally as PLIL-modified black TiO_2_; its coloration should not be attributed to Ti^3+^, oxygen vacancies, or other specific defect states because these were not directly characterized.

Within the investigated 0.1–1.0 wt% regime, TiO_2_ incorporation produced a statistically significant but moderate and formulation-dependent increase in Vickers microhardness. The highest value was obtained with 1.0 wt% untreated white TiO_2_ (4.19 ± 0.12 HV0.05), approximately 25% above neat UHMWPE. The tribological response was non-monotonic: 0.5 wt% PLIL-modified black TiO_2_ exhibited the lowest measured mean wear volume (2.06 ± 0.02 mm^3^), approximately 13.1% below neat UHMWPE, although this difference was not statistically significant after Dunnett adjustment (*p* = 0.739).

Maximum microhardness, minimum terminal friction, and minimum measured mean wear occurred in different formulations, demonstrating that these responses are partially decoupled and that neither hardness nor friction alone predicts wear performance. The results establish a processing–structure–response relationship in which PLIL-induced modification of TiO_2_ and nanoparticle concentration jointly influence UHMWPE tribomechanical behavior. The findings are restricted to the present dry-sliding screening conditions and should not be interpreted as direct evidence of improved in-service orthopedic performance.

## Figures and Tables

**Table 1:** Volumetric wear loss and specific wear rate of UHMWPE nanocomposites containing untreated white TiO_2_.

Material	Wear Volume (mm^3^)	Specific Wear Rate (×10^−4^ mm^3^ N^−1^ m^−1^)
Neat UHMWPE	2.37 ± 0.23	1.49 ± 0.14
0.1 wt% white TiO_2_	2.63 ± 0.24	1.65 ± 0.15
0.3 wt% white TiO_2_	2.70 ± 0.61	1.70 ± 0.38
0.5 wt% white TiO_2_	2.57 ± 0.17	1.62 ± 0.11
0.8 wt% white TiO_2_	2.30 ± 0.04	1.45 ± 0.03
1.0 wt% white TiO_2_	2.36 ± 0.19	1.48 ± 0.12

**Table 2:** Volumetric wear loss and specific wear rate of UHMWPE nanocomposites containing PLIL-modified black TiO_2_.

Material	Wear Volume (mm^3^)	Specific Wear Rate (×10^−4^ mm^3^ N^−1^ m^−1^)
Neat UHMWPE	2.37 ± 0.23	1.49 ± 0.14
0.1 wt% black TiO_2_	2.71 ± 0.29	1.70 ± 0.18
0.3 wt% black TiO_2_	2.90 ± 0.39	1.82 ± 0.25
0.5 wt% black TiO_2_	2.06 ± 0.02	1.30 ± 0.01
0.8 wt% black TiO_2_	2.40 ± 0.26	1.51 ± 0.16
1.0 wt% black TiO_2_	2.35 ± 0.25	1.48 ± 0.16

**Table 3:** Mean terminal coefficient of friction (μ_terminal_) of neat UHMWPE and UHMWPE nanocomposites containing untreated white and PLIL-modified black TiO_2_ nanoparticles. For each independent tribological test, μ_terminal_ was calculated over the final 400–530 m of sliding distance (approximately 2040–2700 s). Values are reported as mean ± *SD* from three independently tested specimens (*n* = 3).

Material	μ_terminal,_ (Mean ± *SD* from *n* = 3)
Neat UHMWPE	0.156 ± 0.006
0.1 wt% white TiO_2_	0.120 ± 0.005
0.3 wt% white TiO_2_	0.179 ± 0.007
0.5 wt% white TiO_2_	0.188 ± 0.008
0.8 wt% white TiO_2_	0.130 ± 0.005
1.0 wt% white TiO_2_	0.127 ± 0.006
0.1 wt% black TiO_2_	0.169 ± 0.007
0.3 wt% black TiO_2_	0.192 ± 0.009
0.5 wt% black TiO_2_	0.128 ± 0.006
0.8 wt% black TiO_2_	0.141 ± 0.006
1.0 wt% black TiO_2_	0.124 ± 0.005

**Table 4:** Two-way ANOVA results for the effects of TiO_2_ nanoparticle state and concentration on the mechanical and tribological responses of UHMWPE nanocomposites.

Response	Effect	*F* (df)	*p*-Value	Partial *η*^2^
Microhardness	Nanoparticle state	*F*(_1,20_) = 37.92	<0.0001	0.655
	Concentration	*F*(_4,20_) = 3.68	0.0210	0.424
	State × concentration	*F*(_4,20_) = 1.15	0.3617	0.187
Wear volume	Nanoparticle state	*F*(_1,20_) = 0.068	0.7967	0.003
	Concentration	*F*(_4,20_) = 3.42	0.0277	0.406
	State × concentration	*F*(_4,20_) = 1.36	0.2835	0.214
Mean terminal coefficient of friction (µ_terminal_)	Nanoparticle state	*F*(_1,20_) = 0.704	0.4113	0.034
	Concentration	*F*(_4,20_) = 76.23	<0.0001	0.938
	State × concentration	*F*(_4,20_) = 55.28	<0.0001	0.917

Note: Statistically significant effects (*p* < 0.05) are indicated in bold. Partial *η*^2^ is reported as an estimate of effect size. Neat UHMWPE was excluded from the factorial ANOVA because nanoparticle state is undefined for the unreinforced control.

**Table 5:** Dunnett-adjusted comparisons of TiO_2_/UHMWPE formulations against neat UHMWPE for microhardness, volumetric wear loss, and mean terminal coefficient of friction.

Formulation	Δ Microhardness (HV)	Adjusted *p*	Δ Wear Volume (mm^3^)	Dunnett-Adjusted *p* (Wear)	Δ μ_terminal_	Adjusted *p*
0.1 wt% white TiO_2_	+0.61	<0.001	+0.26	0.868	−0.036	<0.001
0.3 wt% white TiO_2_	+0.49	<0.001	+0.33	0.681	+0.023	0.002
0.5 wt% white TiO_2_	+0.56	<0.001	+0.20	0.966	+0.032	<0.001
0.8 wt% white TiO_2_	+0.73	<0.001	−0.07	>0.999	−0.026	<0.001
1.0 wt% white TiO_2_	+0.85	<0.001	−0.01	>0.999	−0.029	<0.001
0.1 wt% black TiO_2_	+0.31	0.040	+0.34	0.651	+0.013	0.137
0.3 wt% black TiO_2_	+0.38	0.009	+0.53	0.200	+0.036	<0.001
0.5 wt% black TiO_2_	+0.31	0.040	−0.31	0.739	−0.028	<0.001
0.8 wt% black TiO_2_	+0.35	0.017	+0.03	>0.999	−0.015	0.065
1.0 wt% black TiO_2_	+0.48	<0.001	−0.02	>0.999	−0.032	<0.001

Note: Δ represents the estimated difference relative to neat UHMWPE (TiO_2_/UHMWPE—neat UHMWPE). Positive values indicate an increase and negative values indicate a decrease relative to the control. Statistically significant Dunnett-adjusted comparisons are defined at *p* < 0.05.

## Data Availability

The original contributions presented in this study are included in the article. Further inquiries can be directed to the corresponding authors.

## Abbreviations

The following abbreviations are used in this manuscript:

UHMWPE	Ultra-high-molecular-weight polyethylene
PLIL	Pulsed laser irradiation in liquid
HV	Hardness Vickers
IIT	Instrumented indentation tests
